# Lack of the immune adaptor molecule SARM1 accelerates disease in prion infected mice and is associated with increased mitochondrial respiration and decreased expression of NRF2

**DOI:** 10.1371/journal.pone.0267720

**Published:** 2022-05-04

**Authors:** Anne Ward, Forrest Jessop, Robert Faris, Daniel Shoup, Catharine M. Bosio, Karin E. Peterson, Suzette A. Priola

**Affiliations:** 1 Laboratory of Persistent Viral Diseases, Rocky Mountain Laboratories, National Institute of Allergy and Infectious Diseases, Hamilton, Montana, United States of America; 2 Laboratory of Bacteriology, Rocky Mountain Laboratories, National Institute of Allergy and Infectious Diseases, Hamilton, Montana, United States of America; 3 Department of Microbiology and Immunology, Carver College of Medicine, University of Iowa, Iowa City, Iowa, United States of America; Chinese Institute for Brain Research, CHINA

## Abstract

Prion diseases are a group of fatal, transmissible neurodegenerative diseases of mammals. In the brain, axonal loss and neuronal death are prominent in prion infection, but the mechanisms remain poorly understood. Sterile alpha and heat/Armadillo motif 1 (SARM1) is a protein expressed in neurons of the brain that plays a critical role in axonal degeneration. Following damage to axons, it acquires an NADase activity that helps to regulate mitochondrial health by breaking down NAD^+^, a molecule critical for mitochondrial respiration. SARM1 has been proposed to have a protective effect in prion disease, and we hypothesized that it its role in regulating mitochondrial energetics may be involved. We therefore analyzed mitochondrial respiration in SARM1 knockout mice (SARM1^KO^) and wild-type mice inoculated either with prions or normal brain homogenate. Pathologically, disease was similar in both strains of mice, suggesting that SARM1 mediated axonal degradation is not the sole mechanism of axonal loss during prion disease. However, mitochondrial respiration was significantly increased and disease incubation time accelerated in prion infected SARM1^KO^ mice when compared to wild-type mice. Increased levels of mitochondrial complexes II and IV and decreased levels of NRF2, a potent regulator of reactive oxygen species, were also apparent in the brains of SARM1^KO^ mice when compared to wild-type mice. Our data suggest that SARM1 slows prion disease progression, likely by regulating mitochondrial respiration, which may help to mitigate oxidative stress via NRF2.

## Introduction

Prion diseases are fatal, transmissible neurodegenerative diseases of mammals and include Creutzfeldt-Jakob disease in humans, scrapie in sheep, bovine spongiform encephalopathy (BSE) in cattle, and chronic wasting disease (CWD) in cervids. The infectious agents that trigger these diseases are known as prions and are derived from a cell surface glycoprotein that is normally soluble and protease-sensitive known as prion protein (PrP^C^). During prion infection, PrP^C^ undergoes a conformational change to become insoluble, partially protease-resistant, and infectious. This conformationally abnormal form of PrP^C^, termed PrP^Sc^, is thought to be the primary if, not sole, component of the infectious prion. Prion infection is initiated either when the host’s own PrP^C^ molecule undergoes a conformational transition to PrP^Sc^ or when the host is exposed to an exogenous source of prions, usually through ingestion or inoculation. In either case, prions replicate when PrP^Sc^ interacts with PrP^C^ to convert it to more PrP^Sc^. This process can potentially happen in any cell expressing PrP^C^ but primarily occurs in cells of the central nervous and lymphoreticular systems. The primary pathology of prion diseases results from the accumulation of PrP^Sc^ in the brain leading to gliosis, vacuolation, and neuronal loss, which results in the characteristic spongiform appearance of the brain during the clinical stage of disease.

The mechanisms underlying neurodegeneration in prion diseases are poorly understood. Multiple pathways have been implicated including chronic activation of the unfolded protein response, protein aggregate-based inhibition of the ubiquitin proteasome system, and endoplasmic reticulum induced apoptosis (for review see [1]). As with other neurodegenerative diseases triggered by protein misfolding, multiple lines of evidence suggest that disruption of mitochondrial function also contributes to cellular dysfunction and death during prion disease [2]. Mitochondrial pathways of apoptosis have been implicated in late-stage prion disease [3]. Expression of proteins involved in mitochondrial dynamics, the process of fusion and fission that is critical for maintaining a healthy pool of mitochondria, is altered in the brains of mice and hamsters infected with prions [4–10]. Healthy mitochondria are also important in maintaining synaptic maintenance and plasticity, and disruptions to mitochondrial dynamics and energetics have been linked to the loss of synapses and dendrites in the brain [11–13]. Abnormalities in mitochondrial structure consistent with a disruption in mitochondrial dynamics have been observed in prion infected brain, and synaptic and dendritic alterations are pathological features of CJD [14] and also precede axonal loss and neuronal death in rodent prion infection [9, 15–17]. Thus, changes in mitochondrial structure during prion infection are consistent with an impairment of mitochondrial function that could lead to synaptic loss [9] and eventually axonal degeneration and neuronal death.

The disruption of cristae in brain mitochondria of prion infected animals [9, 10] suggests that mitochondrial respiration, the process whereby oxidative phosphorylation leads to the consumption of molecular oxygen and the generation of ATP, may be impaired during prion disease. In the mitochondrial electron transport chain (ETC), there are five protein complexes (CI-CV) in the inner mitochondrial membrane (IMM) which drive redox reactions to produce a proton gradient. Establishment of a proton gradient leads to a membrane potential across the IMM, the energy of which is used to drive the phosphorylation of ADP by Complex V. Changes in the expression level of proteins associated with CI and CV [18] as well as CIV [9] have been reported in rodent models of scrapie while a transcriptome analysis of human CJD brain has shown a decrease in expression for proteins in CI through CV [19]. Biochemically, the enzymatic activity of both CIV cytochrome *c* oxidase and mitochondrial ATPases are significantly decreased during hamster prion infection suggesting impaired mitochondrial respiration [7]. Consistent with this hypothesis, we have recently shown that mitochondrial respiration in response to the CII substrate succinate is significantly decreased during clinical, but not pre-clinical, hamster scrapie infection [18]. Thus, multiple lines of evidence point to impaired mitochondrial respiration during prion infection.

One molecule that localizes to mitochondria and is involved in both synaptic maintenance and axonal degeneration is sterile alpha and heat/Armadillo motif 1 (SARM1), a member of the Toll/IL-1 receptor (TIR) domain family (for review see [20]). SARM1 has a mitochondrial localization signal [21] and can be present on the outer mitochondrial membrane (OMM) where it may be involved in the PINK1/Parkin pathway of mitophagy [22]. In the brain, SARM1 is found in axons and at the synapse [23, 24], where it is likely involved in maintaining synaptic plasticity [25] and neuronal morphogenesis, particularly dendritic arborization [23]. Importantly, SARM1 plays a critical role in axonal degeneration following nerve damage [24, 26] when it’s intrinsic NADase activity is activated, allowing it to cleave nicotinamide dinucleotide (NAD^+^). This leads to a reduction in NAD^+^ and ATP, the result of which is mitochondrial depolarization and axonal degeneration [27, 28]. SARM1 could thus potentially impact prion pathogenesis in multiple ways, including via its interaction with mitochondria and its critical role in axonal degeneration.

A recent study demonstrated that prion infection of SARM1 knockout mice (SARM1^KO^) led to an accelerated disease course which the authors attributed to overexpression of the pro-apoptotic gene *Xaf1* (X-linked inhibitor of apoptosis associated factor 1) and an increase in neuronal apoptosis [29]. We hypothesized that lack of SARM1 might also shorten disease incubation time by exacerbating the deficit in mitochondrial respiration that occurs during prion infection [18]. We therefore analyzed mitochondrial respiration in SARM1^KO^ mice versus wild-type mice inoculated either with RML prions or normal brain homogenate. Our results show that, rather than further depressing mitochondrial respiration, lack of SARM1 actually led to an increase in mitochondrial respiration when compared to prion-infected wild-type mice expressing SARM1. In addition, expression of CII, CIV, and NRF2, a key regulator of oxidative stress [30, 31], was decreased in SARM1^KO^ mice regardless of prion infection. Our data suggest that SARM1 is important in regulating mitochondrial respiration and may influence expression of NRF2 in the brain, functions which could help to mitigate the progression of prion disease and possibly other neurodegenerative protein misfolding diseases.

## Results

### SARM1 exerts a protective effect during prion infection

**Fig 1:**
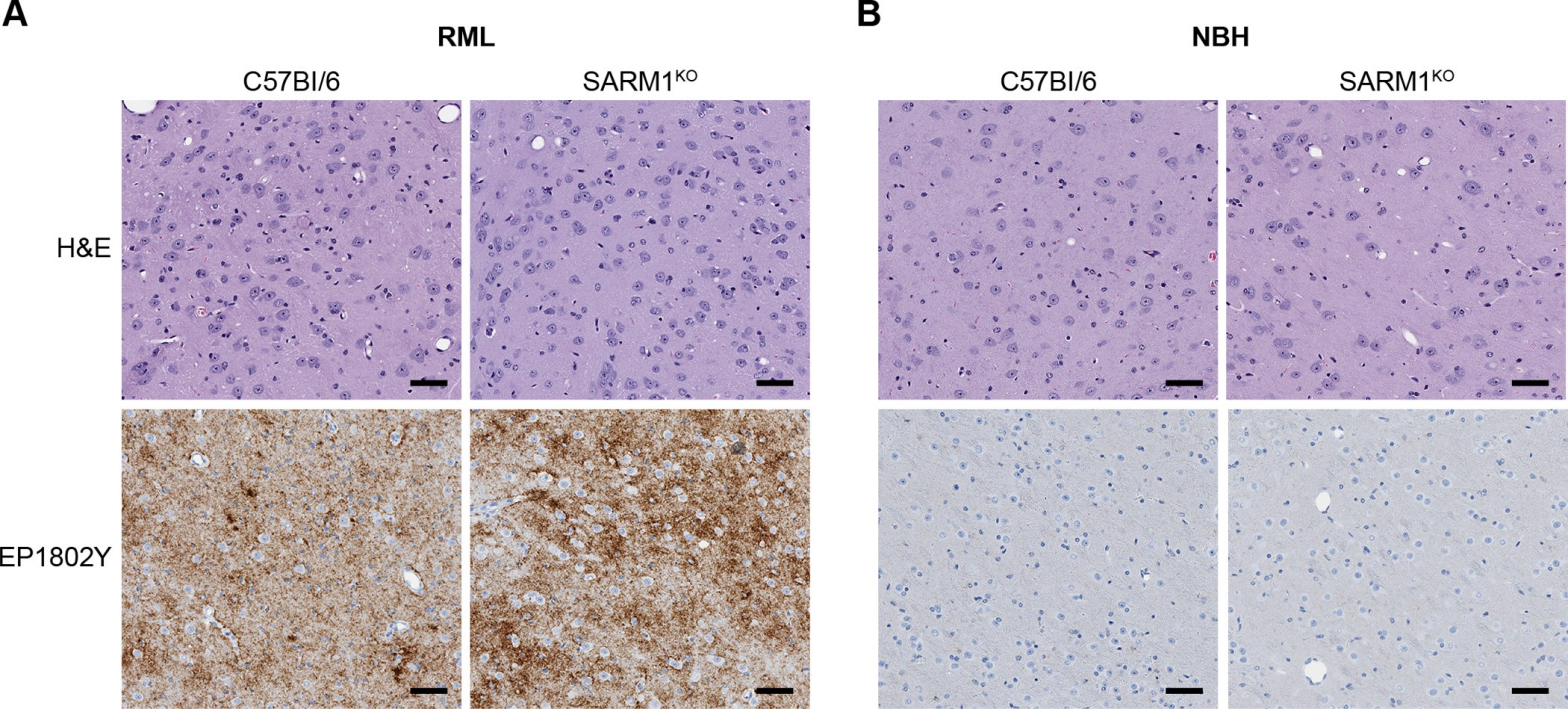
PrP^Sc^ deposition and spongiform change in the brain are similar in SARM1^KO^ and C57Bl/6 mice 75 days after intracranial inoculation with RML prions. (A) H&E and PrP^Sc^ staining for C57Bl/6 (left panels) and SARM1^KO^ (right panels) in the thalamus of mice 75 DPI after inoculation intracranially with RML prions. (B) H&E and PrP^Sc^ staining for C57Bl/6 (left panels) and SARM1^KO^ (right panels) in the thalamus of mice 75 DPI after inoculation intracranially with normal brain homogenate (NBH). PrP^Sc^ was detected using the anti-PrP rabbit monoclonal antibody EP1802Y as described in the materials and methods. For all panels, scale bar = 50μm.

C57Bl/6 and SARM1^KO^ mice were inoculated intracranially (IC) with the RML mouse prion strain. PrP^Sc^ deposition and spongiform change were assayed at 75 days post-infection (DPI), the approximate midpoint of the incubation period of RML prions in C57Bl/6 mice. At 75 days, PrP^Sc^ was detectable in the brains of both mouse lines primarily in the thalamus. PrP^Sc^ was deposited in a diffuse, punctate/synaptic pattern in both strains of mice with no consistent differences in the intensity of PrP^Sc^ deposition (Fig 1A). Neuropathologically, RML inoculated mice were similar to negative control mice inoculated with normal brain homogenate (NBH) and no spongiform change was apparent in either mouse strain (Fig 1B). Thus, lack of SARM1 did not appear to have any obvious impact on the early progression of prion infection.

**Fig 2:**
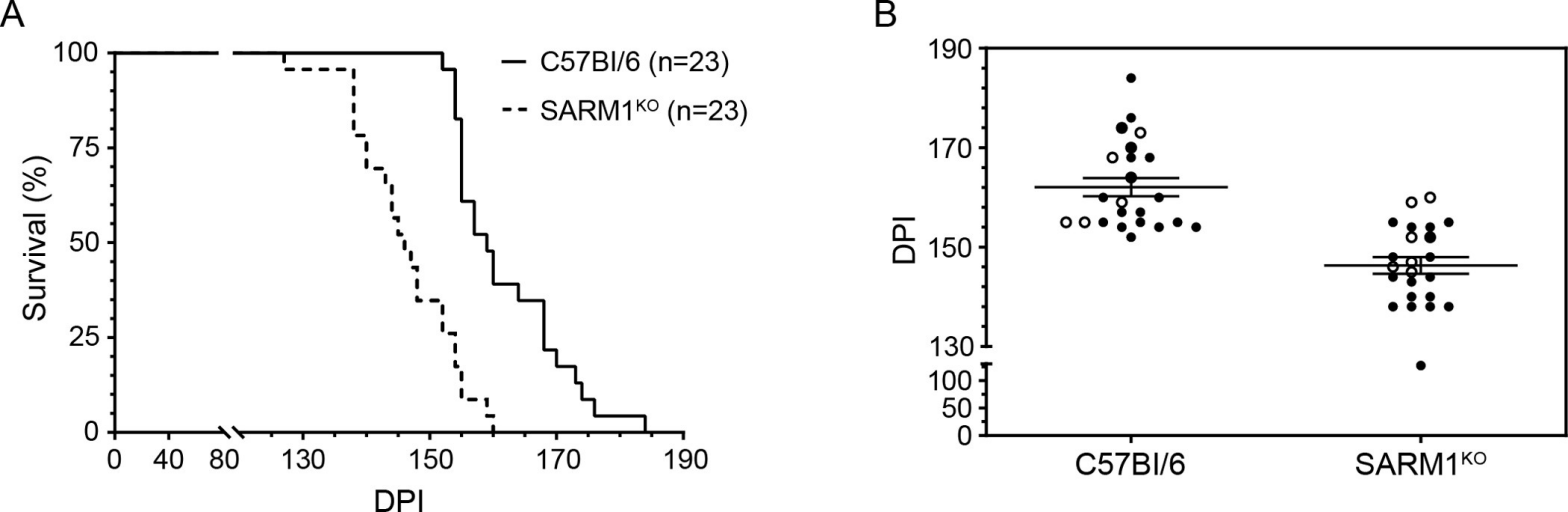
Decreased disease incubation times in SARM1^KO^ mice infected with RML prions. (A) Survival curves for C57Bl/6 (solid line) and SARM1^KO^ (dotted line) mice inoculated intracranially with RML prions. The number of mice inoculated is given in parentheses. (B) Scatter plot for SARM1^KO^ and C57Bl/6 mice inoculated intracranially with RML prions. Each circle represents one mouse and open circles indicate mice used in the Seahorse XFe96 mitochondrial coupling assay. Mean incubation times plus standard error of the mean (SEM) are indicated and are 162 ± 1.9 days for C57Bl/6 mice and 146 ± 1.7 days for SARM1^KO^ mice. Data are pooled from four independent experiments. Intercurrent deaths were excluded. Disease incubation times for C57Bl/6 and SARM1^KO^ mice differed significantly (****, p < 0.0001, unpaired Student’s t-test). DPI = days post-infection.

**Fig 3:**
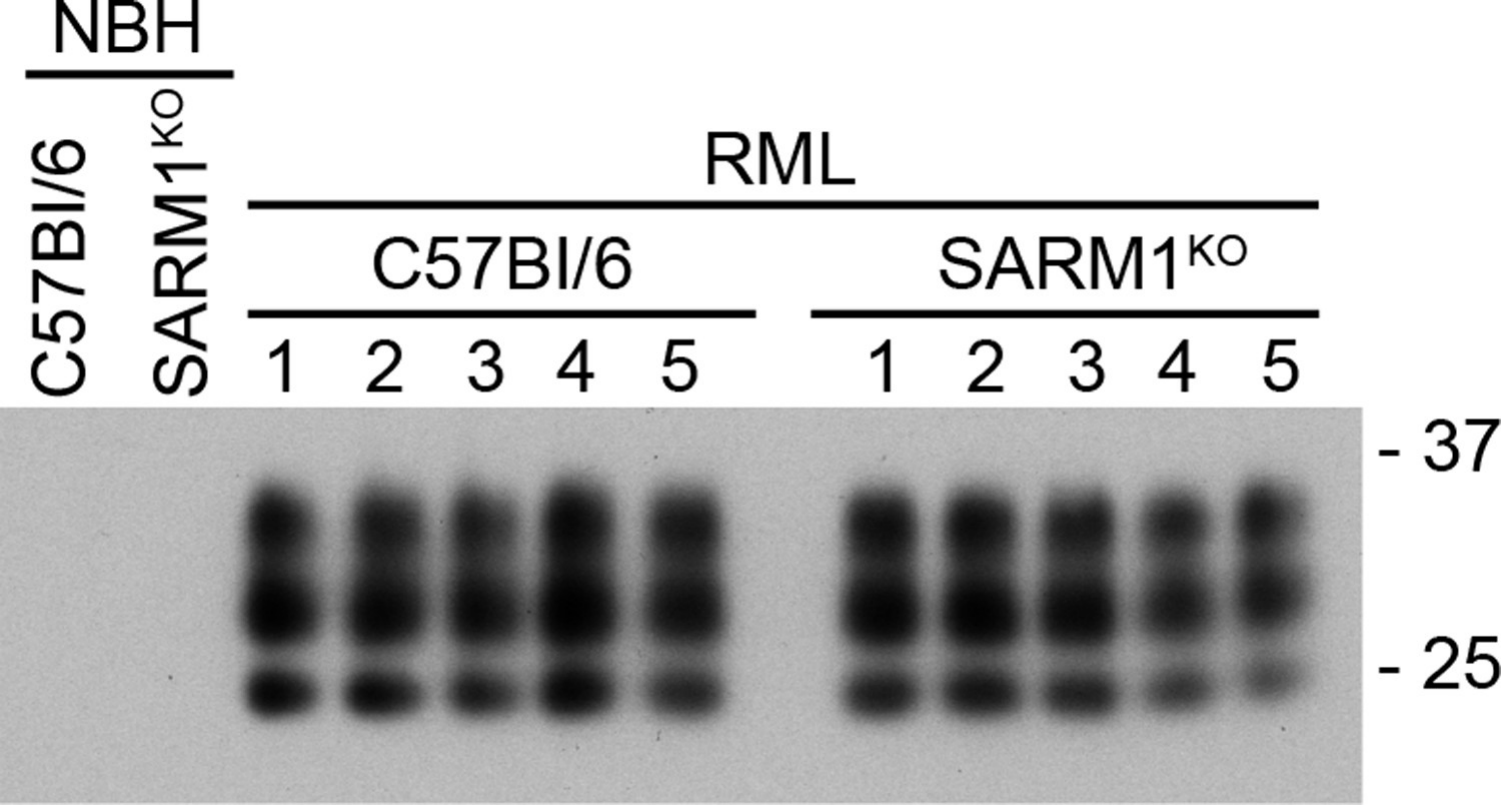
SARM1^KO^ and C57Bl/6 mice infected with RML prions accumulate similar levels of PrP^Sc^ in the brain. Western blot analysis of PrP^Sc^ in the brains of C57Bl/6 and SARM1^KO^ mice inoculated with RML prions (RML) or normal brain homogenate (NBH). All samples were PK-treated as described in the materials and methods. The sample number is given above each lane. As a negative control, the first two lanes show a single C57Bl/6 or SARM1^KO^ mice inoculated with NBH. Unlabeled lanes are empty. The blot was developed using the anti-PrP mouse monoclonal antibody 6D11. Molecular mass markers in kilodaltons are shown on the right.

**Fig 4:**
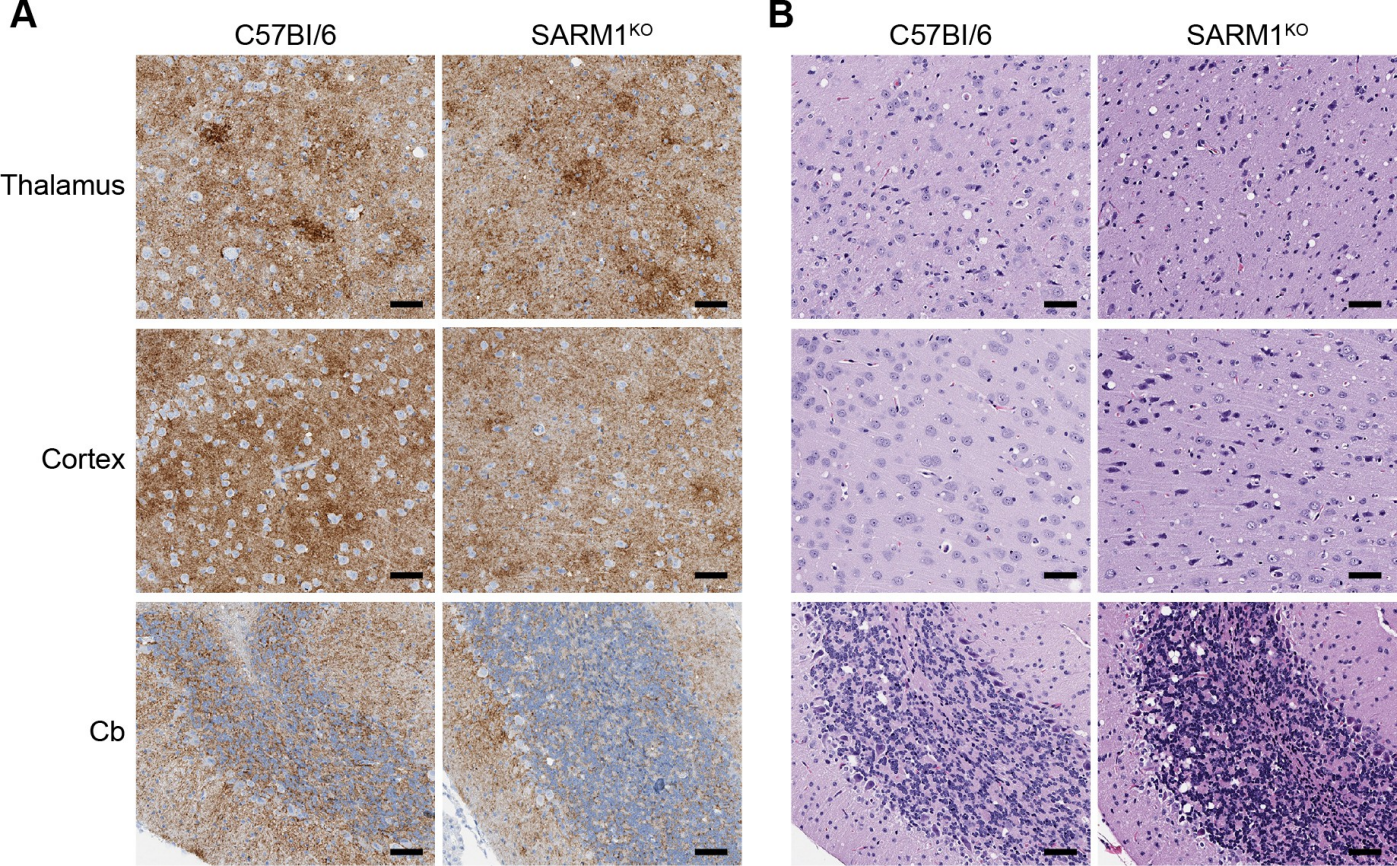
The pattern of PrP^Sc^ deposition and spongiform change in the brain are similar at the clinical stage of disease in SARM1^KO^ and C57Bl/6 mice inoculated IC with RML prions. (A) PrP^Sc^ staining in the thalamus, cortex, and cerebellum (Cb) of clinically positive C57Bl/6 (155 DPI, left panels) and SARM1^KO^ (148 DPI, right panels) mice inoculated IC with RML prions. PrP^Sc^ was detected using the anti-PrP rabbit monoclonal antibody EP1802Y as described in the materials and methods. (B) H&E staining of the thalamus, cortex, and cerebellum from clinically positive C57Bl/6 (155 DPI, left panels) and SARM1^KO^ (148 DPI, right panels) mice inoculated intracranially with RML prions. For all panels, scale bar = 50μm.

Despite the similarities in PrP^Sc^ deposition at 75 days, SARM1^KO^ mice progressed to clinical disease more rapidly than C57Bl/6 mice. The results are summarized in the survival and scatter plots shown in Fig 2. The average incubation time to clinical disease of RML prions in SARM1^KO^ mice was 146 ± 1.7 dpi versus 162 ± 1.9 dpi for C57BL/6 mice, a highly significant difference of p < 0.0001. At clinical disease, a diffuse, punctate/synaptic pattern of PrP^Sc^ deposition was seen in both SARM1^KO^ and C57Bl/6 mice. PrP^Sc^ was widespread throughout the brain including the thalamus, cortex, and cerebellum. There was no discernible difference in the amount (Fig 3 and S1A Fig), pattern, or intensity of PrP^Sc^ deposition between the two mouse strains (Fig 4A). Similarly, there was no difference neuropathologically between RML-infected C57Bl/6 and SARM1^KO^ mice, with spongiform change widespread throughout the brain including in the thalamus, cortex, and cerebellum (Fig 4B and S1B Fig). Our results are consistent with a previous study of prion infection of SARM1^KO^ mice [29] and suggest that SARM1 exerts a protective effect during prion infection that does not correlate with PrP^Sc^ accumulation or the development of spongiform change.

### Similar rates of mitochondrial respiration in aged SARM1^KO^ and C57Bl/6 mice

We have previously shown that mitochondrial respiration initiated in response to the CII substrate succinate is significantly impaired in hamsters infected with prions [18]. The protective effect of SARM1 during prion infection could be related to its role in maintaining mitochondrial health. Thus, a lack of SARM1 might exacerbate the deficit in mitochondrial respiration that can occur during prion infection [18].

**Fig 5:**
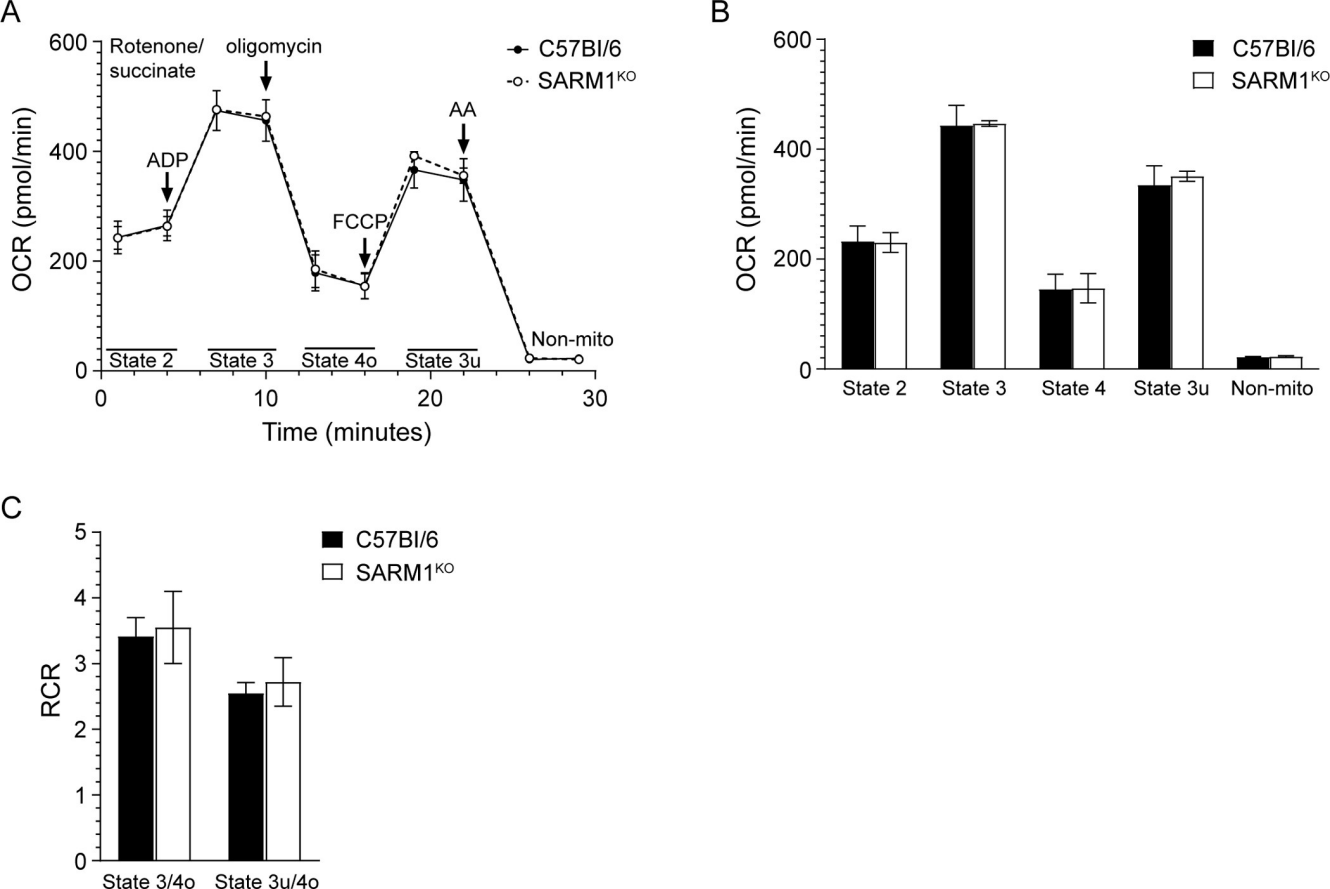
No difference in mitochondrial respiration in aged SARM1^KO^ and C57Bl/6 mice. Mitochondrial coupling assay measuring mitochondrial respiration for aged C57Bl/6 (n = 3) and SARM1^KO^ (n = 3) mice. (A) Oxygen consumption rates (OCR) for C57Bl/6 (black circle, solid line) and SARM1^KO^ (open circle, dotted line) mice. The reagents added during the 30 min assay as well as the states quantified are indicated. AA = antimycin A; Non-mito = non mitochondrial respiration. (B) OCRs and (C) RCRs for the data in Panel A. Values were calculated as described in the materials and methods. Black bars = C57BL/6, white bars = SARM1^KO^. For all panels, the mean ± SEM is shown.

We first determined if a lack of SARM1 affects mitochondrial respiration in the absence of prion replication. Mitochondria were isolated from aged SARM1^KO^ and C57Bl/6 mice and mitochondrial respiration was analyzed using the Seahorse XFe96 Analyzer. Following inhibition of CI with rotenone and the addition of saturating amounts of ADP, succinate was used as a substrate to stimulate mitochondrial respiration via CII. For each assay, a single C57Bl/6 and a single SARM1^KO^ mouse were analyzed on the same plate. Although there was some variability between the mouse pairs assayed (S1 Table), the oxygen consumption rate (OCR) of aged SARM1^KO^ and C57Bl/6 mice was similar (Fig 5A). When the data from 3 individual mice were combined, there were no significant differences in the OCR of State 2 (basal respiration), State 3 (phosphorylating respiration), State 4o (proton leak), State 3u (oxidative capacity) and non-mitochondrial respiration (Fig 5B). The respiratory control ratios (RCR) of both state 3/state 4o and state 3u/4o, which indicate how tightly mitochondrial respiration and phosphorylation are coupled, were identical between the mouse strains (Fig 5C and S1 Table). Overall, the calculated RCRs were consistent with previous analyses of the CII RCR of brain mitochondria [32] and suggested that the mitochondria were functional and intact, with good coupling of mitochondrial respiration and phosphorylation. Thus, in healthy mice, lack of SARM1 does not appear to have a significant impact on the ability of mitochondria to consume oxygen and generate ATP.

### Mitochondrial respiration is not significantly impaired in RML prion infected C57Bl/6 mice

In order to determine the impact of prion infection on mitochondrial respiration in wild-type mice, mitochondria were isolated from individual C57Bl/6 mice at the clinical stage of prion infection (open circles, Fig 2B) as well as from control C57Bl/6 mice inoculated with NBH. Mitochondrial respiration was monitored using the assay conditions for aged mice described above. For each assay, mitochondria from a single RML prion infected mouse and a single NBH inoculated mouse were analyzed on the same plate.

**Fig 6:**
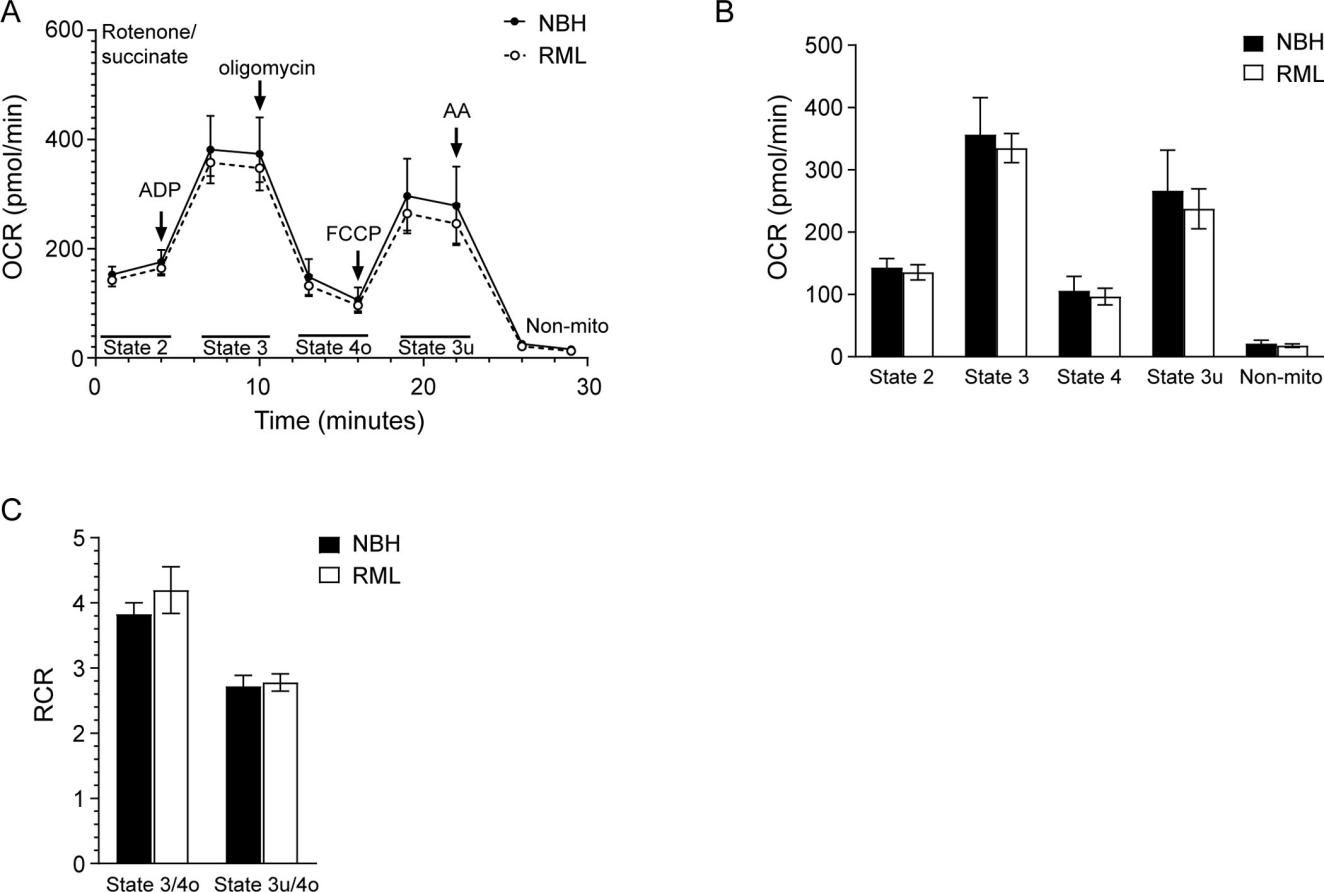
No difference in mitochondrial oxygen consumption in C57Bl/6 mice infected with RML prions. (A) Mitochondrial coupling assay measuring mitochondrial respiration for clinically positive C57Bl/6 mice infected with RML prions (open circle, dotted line) or inoculated with NBH (black circle, solid line). A total of n = 5 mice were assayed for each condition. The reagents added during the 30 min assay as well as the states quantified are indicated. AA = antimycin A; Non-mito = non mitochondrial respiration. (B) OCRs and (C) RCRs for the data in Panel A. Values were calculated as described in the materials and methods. Black bars = NBH inoculated, white bars = RML inoculated. For all panels, the mean ± SEM is shown.

As shown in Fig 6A, the OCR was slightly lower in C57Bl/6 mice infected with RML prions with the results somewhat variable from assay to assay (S2 Table). When the data from 5 biological replicates was combined, there was no significant difference in the OCRs of any of the states when compared to the corresponding NBH inoculated control (Fig 6B). RCR values for state 3/state 4o and state 3u/4o were also similar between prion and NBH inoculated mice, indicating that the IMM was intact with good coupling of mitochondrial respiration and phosphorylation (Fig 6C). The data suggest that, unlike in hamster prion infection [18], there is no significant effect on mitochondrial oxygen consumption rate during mouse prion infection.

### Mitochondrial respiration in RML prion infected SARM1^KO^ mice

**Fig 7:**
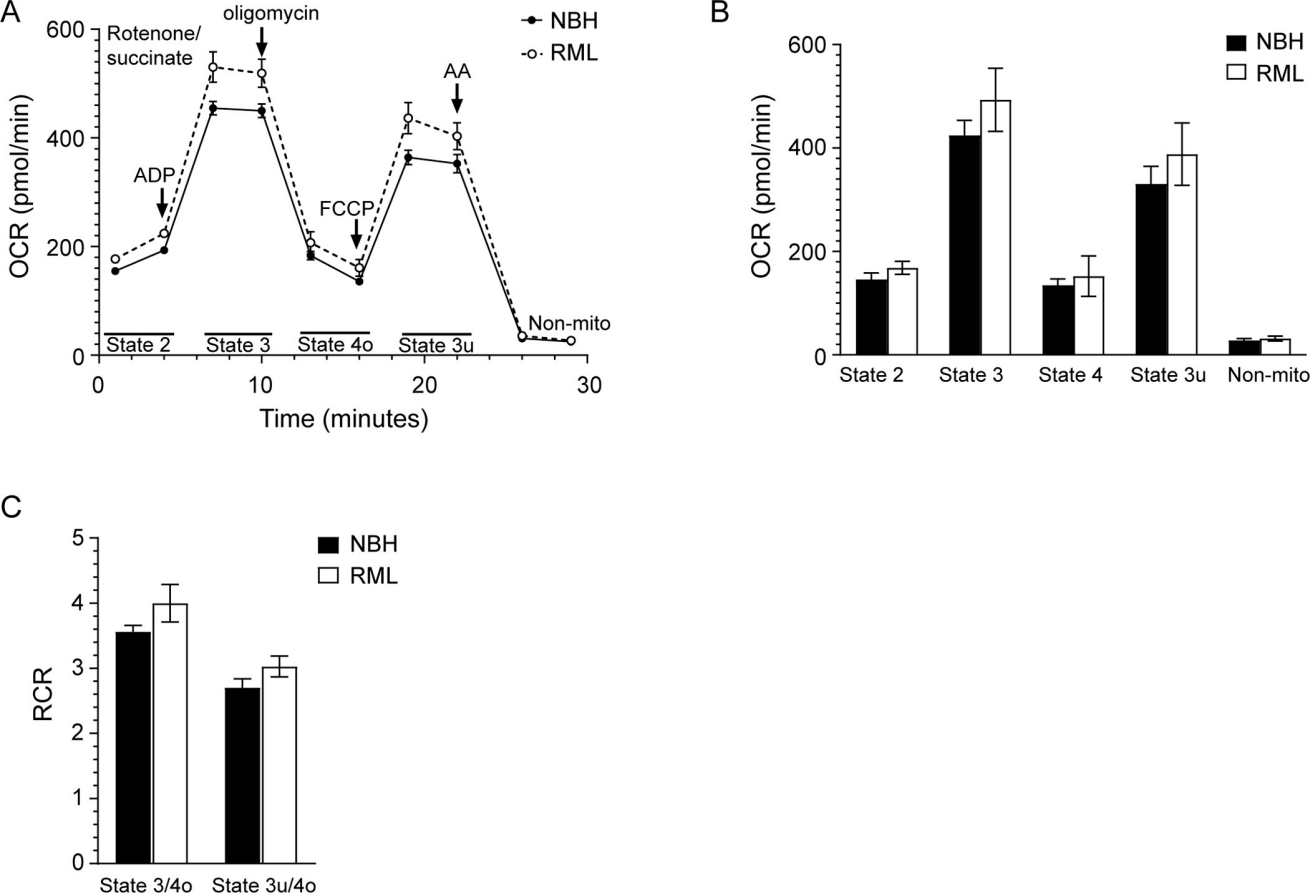
No significant difference in mitochondrial oxygen consumption in SARM1^KO^ mice infected with RML prions. (A) Mitochondrial coupling assay measuring mitochondrial respiration for clinically positive SARM1^KO^ mice infected with RML prions (open circle, dotted line) or inoculated with NBH (black circle, solid line). A total of n = 6 mice were assayed for each condition. The reagents added during the 30 min assay as well as the states quantified are indicated. AA = antimycin A; Non-mito = non mitochondrial respiration. (B) OCRs and (C) RCRs for the data in Panel A. Values were calculated as described in the materials and methods. Black bars = NBH inoculated, white bars = RML inoculated. For all panels, the mean ± SEM is shown.

We next determined the impact of prion infection on mitochondrial respiration in SARM1^KO^ mice infected with RML prions. Mitochondria were isolated from individual SARM1^KO^ mice at the clinical stage of prion infection (open circles, Fig 2B) as well as from control SARM1^KO^ mice inoculated with NBH, and mitochondrial respiration in response to succinate analyzed. As shown in Fig 7A, the OCR appeared to be higher in SARM1^KO^ mice infected with RML prions when compared to NBH inoculated controls (Fig 7A). In particular, the State 2 and 3 OCRs were significantly higher in the majority of SARM1^KO^ mice tested when compared to NBH inoculated control mice (S3 Table). The average lower OCR observed in NBH inoculated SARM1^KO^ mice was not the result of disruption to mitochondrial function as the RCR values for state 3/state 4o and state 3u/4o were similar between prion and NBH inoculated SARM1^KO^ mice, indicating that the IMM was intact and that mitochondrial respiration and phosphorylation were coupled (Fig 7C). However, when OCRs were calculated from the 6 combined biological replicates following subtraction of non-mitochondrial respiration (see materials and methods), there was no significant difference in the OCRs of State 3 or any of the other states when compared to the corresponding NBH inoculated SARM1^KO^ control (Fig 7B). This lack of significance was likely due to the relatively wide variability in the calculated OCRs from assay to assay (S3 Table).

**Fig 8:**
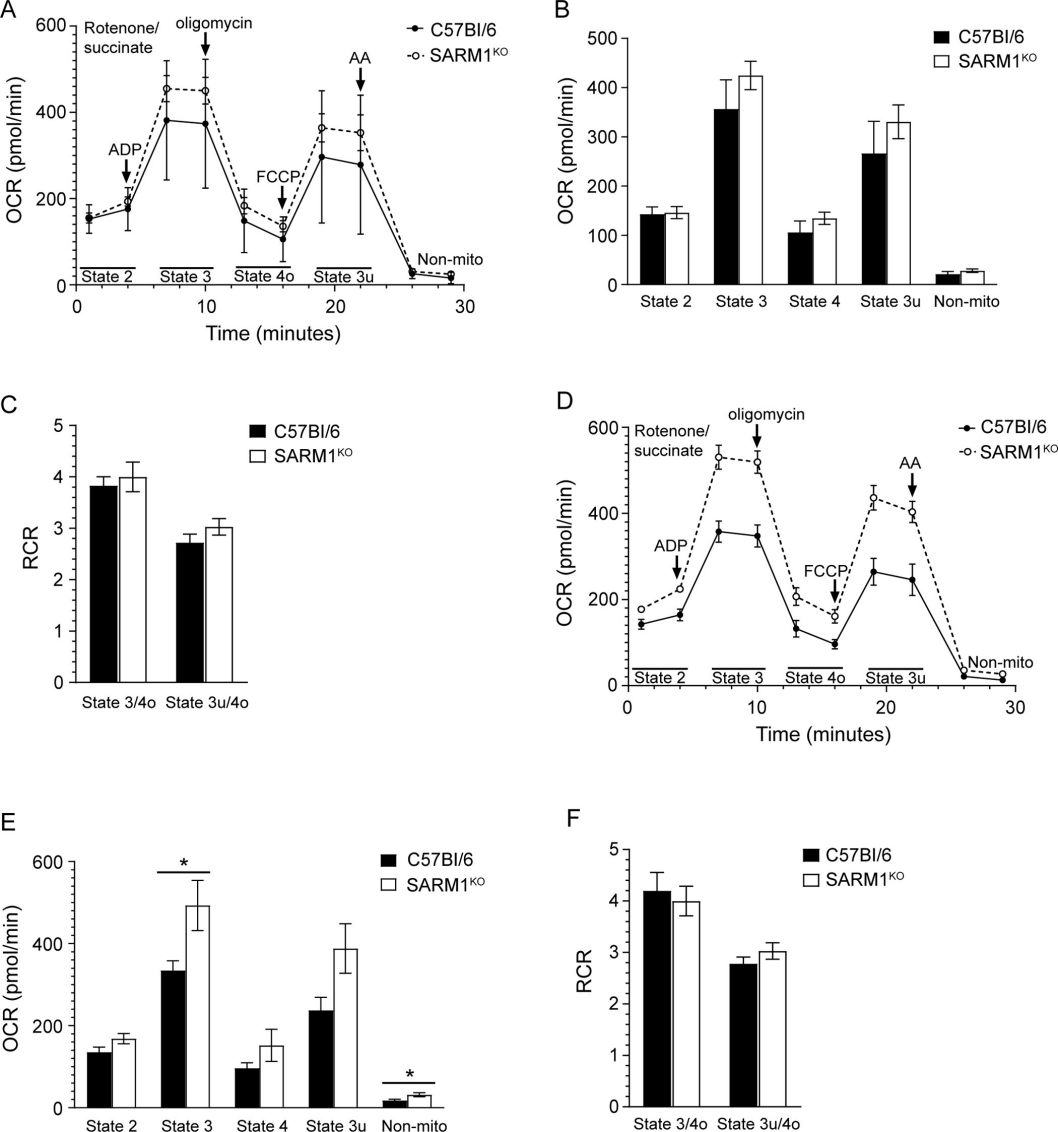
Increased State 3 OCR in RML prion infected SARM1^KO^ mice compared to RML prion infected C57Bl/6 mice. (A) Mitochondrial coupling assay measuring mitochondrial respiration for C57Bl/6 (n = 5; black circle, solid line) and SARM1^KO^ (n = 6; open circle, dotted line) mice inoculated with NBH. The reagents added during the 30 min assay as well as the states quantified are indicated. AA = antimycin A; Non-mito = non mitochondrial respiration. (B) OCRs and (C) RCRs for the data in Panel A. Data were calculated as described in the materials and methods. Black bars = C57BL/6, white bars = SARM1^KO^. (D) Mitochondrial coupling assay measuring mitochondrial respiration for C57Bl/6 (n = 5; black circle, solid line) and SARM1^KO^ (n = 6; open circle, dotted line) mice infected with RML prions. (B) OCRs and (C) RCRs for the data in Panel D. Values were calculated as described in the materials and methods. Black bars = C57BL/6, white bars = SARM1^KO^. For all panels, data are derived from Figs 6 and 7, with the mean ± SEM shown. Statistical significance was calculated using the unpaired Student’s t-test with Welch’s correction. * p = 0.05.

For the majority of mice, the OCRs in prion infected SARM1^KO^ mice were higher than those in prion infected wild-type mice (S2 and S3 Tables), suggesting that mitochondrial respiration might be increased in prion infected SARM1^KO^ mice relative to C57BL/6 mice. Direct comparison of the OCRs between RML prion infected SARM1^KO^ and C57Bl/6 mice confirmed that the OCR was higher in the SARM1^KO^ mice (Fig 8D). Importantly, the OCR of State 3 was significantly higher in the SARM1^KO^ mice, while the OCR of State 3u was just outside of the cutoff point for significance (Fig 8E, p = 0.06). The rate of non-mitochondrial respiration was also slightly but significantly higher in RML prion infected SARM1^KO^ mice (Fig 8E). However, this would not contribute to a significantly higher State 3 OCR since non-mitochondrial respiration is subtracted when calculating the OCRs of the different states. In contrast to RML infected mice, there were no significant differences in the OCRs of the different states of NBH inoculated SARM1^KO^ mice when compared to wild-type mice (Fig 8A and 8B). Moreover, the RCRs were identical for both strains of mice whether inoculated with NBH or prions (Fig 8C and 8F) indicating that the mitochondria were healthy. Overall, the data are consistent with the significant increase in State 3 OCR in SARM1^KO^ versus C57Bl/6 mice being a consequence of prion infection and suggest that SARM1 may help to regulate mitochondrial respiration, with its absence leading to higher rates of oxygen consumption during prion infection.

### Increased levels of mitochondrial proteins in RML prion infected C57Bl/6 mice

**Fig 9:**
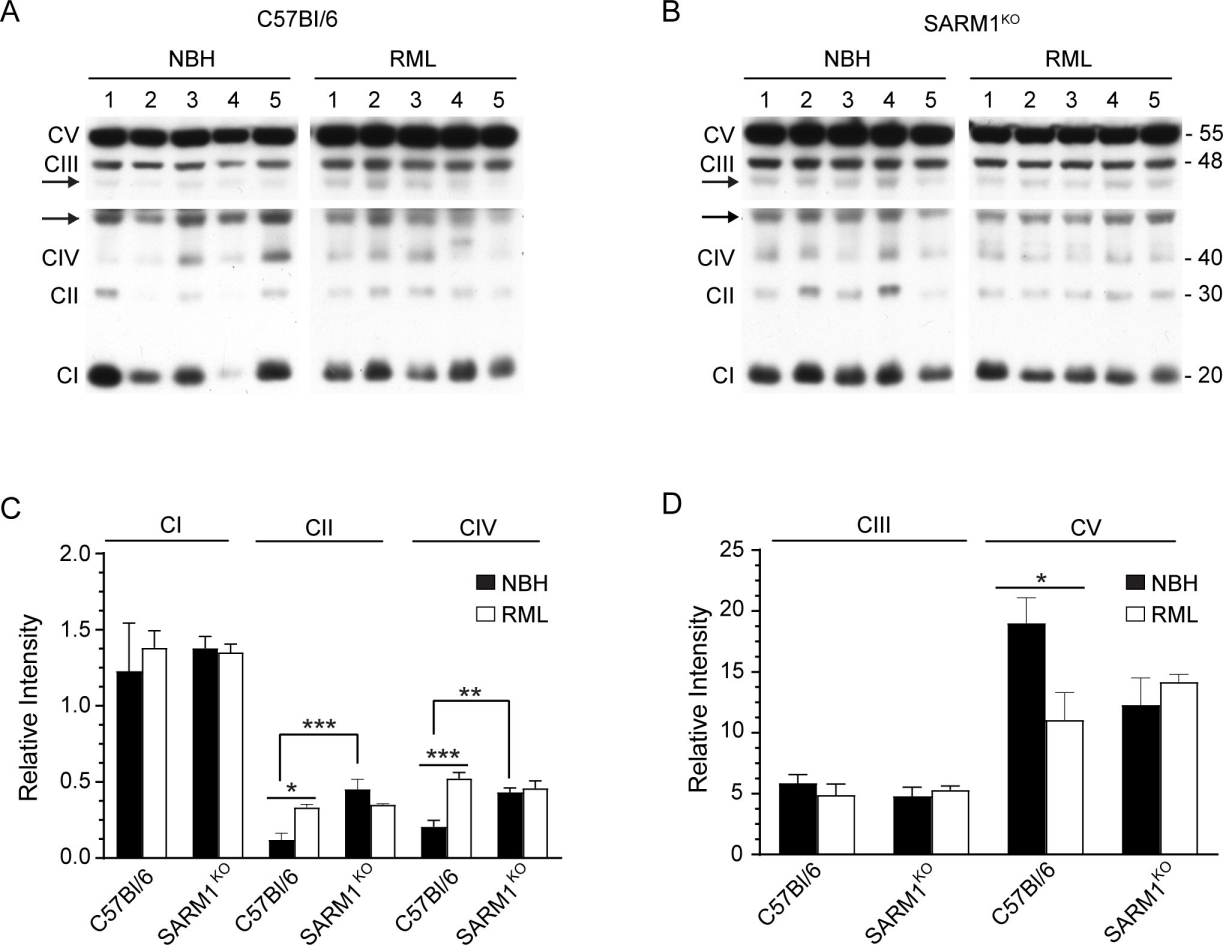
Altered expression levels of mitochondrial complex proteins in prion infected mice. (A) Expression level of mitochondrial complexes CI-CV in C57Bl/6 mice inoculated with NBH or RML prions. (B) Expression of mitochondrial complexes CI-CV in SARM1^KO^ mice inoculated with NBH or RML prions. For both A and B, blots were developed using the Total OXPHOS Rodent WB Antibody Cocktail and an antibody to β-actin. The upper and lower panels are different exposures of the same blot, with the 45 kDa β-actin band indicated by the black arrows on the left side of the panel. Numbers above each lane represent individual mice. The molecular mass in kDa of the proteins detected in CI-CV is indicated on the right side of Panel B. Quantitation of (C) expression levels of CI, CII, and CIV and (D) expression levels of CIII and CV. Data were normalized to mouse β-actin (Relative Intensity) and were calculated from n = 5 animals for each condition. Black bars = NBH inoculated, open bars = RML prion inoculated. Mean ± SEM is shown. Significance was calculated using a 1-way ANOVA multiple comparisons test with NBH inoculated C57Bl/6 mice set as the control. * p = 0.01–0.03; ** p = 0.003; *** p = 0.0001–0.0002.

Changes in the expression levels of CI-CV have been reported in prion infected brain [9, 18, 19], suggesting that mitochondrial respiration is altered during prion infection. Thus, the significant increase in mitochondrial oxygen consumption that we observed in prion infected SARM1^KO^ mice versus C57Bl/6 mice could be due to changes in the expression levels of Complexes I-V. We therefore analyzed the levels of key proteins in each of the 5 mitochondrial complexes in the brains of inoculated C57Bl/6 (Fig 9A) and SARM1^KO^ (Fig 9B) mice. For both mouse strains, there was no difference in the amount of CI (Fig 9C) and CIII (Fig 9D) when NBH inoculated mice were compared to RML inoculated mice. Consistent with previous studies [9, 18, 19], CII and CIV were significantly elevated and CV significantly decreased in prion infected C57Bl/6 mice when compared to NBH inoculated controls (Fig 9C and 9D), indicating prion-related changes in mitochondrial function. Interestingly, expression levels of CII and CIV were already significantly elevated in uninfected SARM1^KO^ mice compared to C57BL/6 mice (Fig 9C) suggesting that, even in the absence of prion infection, mitochondrial homeostasis is altered in these mice.

### Altered mitochondrial dynamics in RML prion infected C57Bl/6 mice and SARM1^KO^ mice

**Fig 10:**
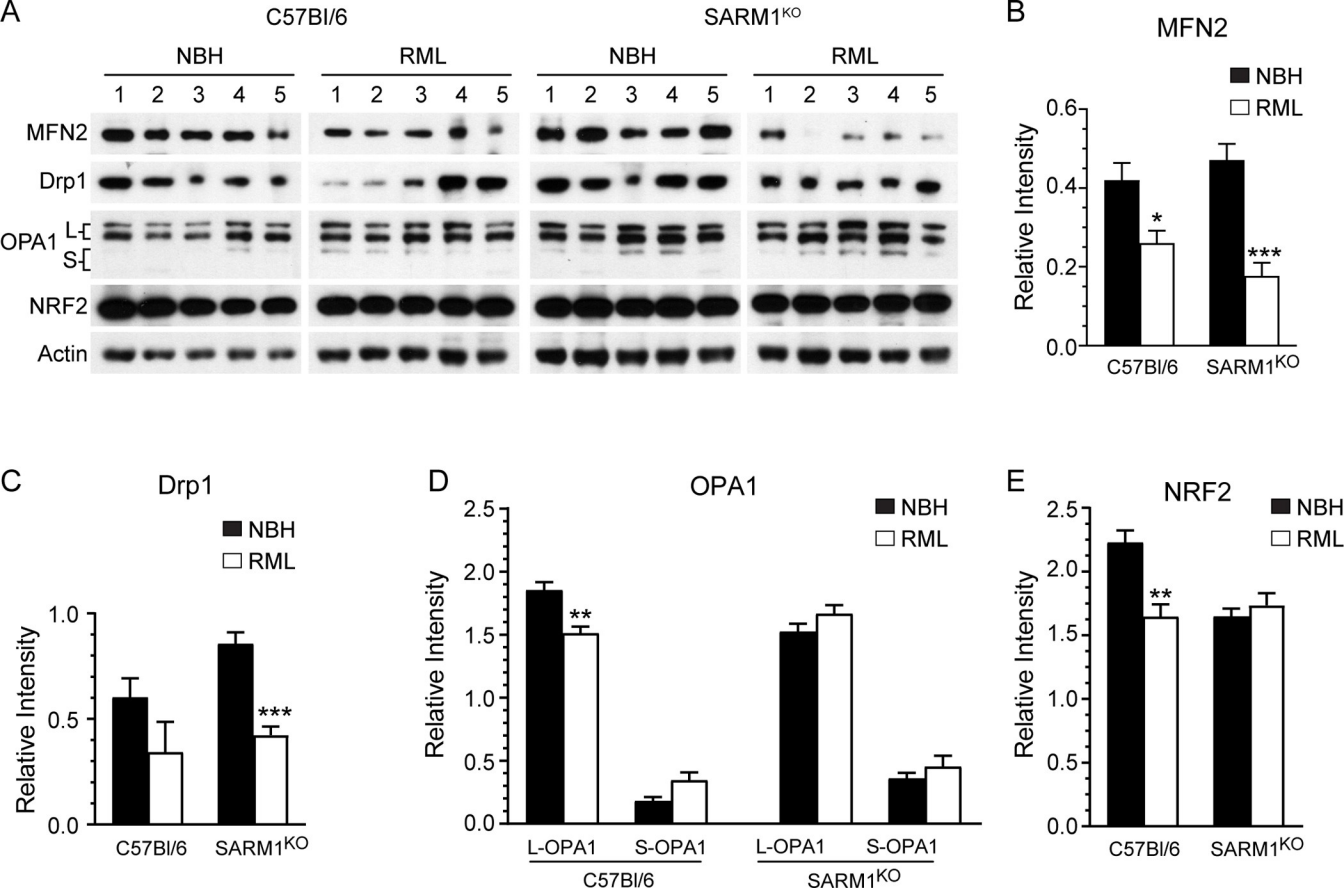
Decreased expression of proteins involved in mitochondrial dynamics and regulation of oxidative stress in prion infected mice. (A) Representative immunoblots for MFN2, Drp1, OPA1, NRF2 and mouse β-actin. Data are from C57Bl/6 (left two panels) or SARM1^KO^ mice (right two panels) inoculated IC with normal brain homogenate (NBH) or RML prions (RML). The brackets on the far left indicate the two bands representing L-OPA1 (L) and the two bands representing S-OPA1 (S). The antibodies used are described in the materials and methods. Quantitation of (B) MFN2, (C) Drp-1, (D) OPA1 long (L-OPA1) and short (S-OPA1), and (E) NRF2 in C57Bl/6 and SARM1^KO^ mice inoculated with NBH or RML prions. Data were normalized to mouse β-actin (Relative intensity) and were calculated from n = 5 animals for each condition. For panels B-E, mean ± SEM is shown. Significance was calculated using the unpaired Student’s t-test with Welch’s correction. * p = 0.02; ** p = 0.003–0.004; *** p = 0.0003–0.0006. Black bars = NBH inoculated, open bars = RML prion inoculated.

Mitochondrial health is maintained via a constant process of mitochondrial fission and fusion, which is used to remove damaged mitochondria via mitophagy as well as to increase the numbers of healthy mitochondria when physiologically necessary. This process, known as mitochondrial dynamics, is often disrupted during infection or disease, including during prion disease [5]. We therefore analyzed the levels of several proteins involved in the process of mitochondrial fusion and fission (Fig 10A) to determine if impaired mitochondrial dynamics correlated with the faster disease incubation times observed in prion infected SARM1^KO^ mice. Mitofusion 2 (MFN2), a key protein in the process of mitochondrial fusion, was significantly decreased in prion infected C57Bl/6 and SARM1^KO^ mice, with no difference in MFN2 levels between the two mouse strains (Fig 10B). Similarly, the expression level of dynamin related protein-1 (Drp-1), a protein involved in mitochondrial fission that has been shown to be decreased during prion infection [5, 6], was also down in both prion infected C57Bl/6 and SARM1^KO^ mice (Fig 10C). The decrease in Drp-1 expression was clearly significant in the prion infected SARM1^KO^ mice (p = 0.007) but was just outside the cutoff for significance in C57Bl/6 mice (p = 0.052).

Finally, we looked at the level of optical atrophy protein 1 (OPA1), a mitochondrial dynamin-like GTPase which mediates fusion of the IMM [33]. Cleavage of the long 120kDa forms of OPA1 (L-OPA1) into shorter forms known as S-OPA1 are associated with increased stress and mitochondrial fission [34]. While OPA1 expression is decreased during prion infection [5], it is unclear how cleavage of OPA1 is affected. We therefore analyzed the expression levels of L-OPA1 and S-OPA1 to determine whether or not OPA1 was being processed differently in C57Bl/6 versus SARM1^KO^ mice. L-OPA1 was significantly decreased in prion infected C57Bl/6 mice (Fig 10D, p = 0.004) while S-OPA1 appeared to be increased, although this increase was not significant (p = 0.06). There was no difference in the expression levels of either form of OPA1 in SARM1^KO^ prion infected mice when compared to uninfected, NBH inoculated control mice (Fig 10D). Overall, our data suggest similarly altered mitochondrial dynamics in both C57Bl/6 and SARM1^KO^ prion infected mice, with the only difference between the two mouse strains being a slight but significant decrease in L-OPA1 levels in C57Bl/6 mice expressing SARM1.

### Decreased levels of NRF2 in SARM1^KO^ mice

The transcription factor NRF2 is an important regulator of cellular defenses against oxidative stress [30, 31] and its activation can lead to increased rates of mitochondrial respiration [35]. NRF2 has been reported to bind PrP [36] and during prion infection its expression can be decreased [36, 37] or increased [38]. We therefore analyzed NRF2 expression in the presence or absence of prion infection in SARM1^KO^ and wild-type mice. Unlike in human CJD [38], NRF2 levels were significantly decreased in RML prion infected C57Bl/6 mice when compared to NBH inoculated controls (Fig 10E). Interestingly, the expression level of NRF2 in NBH inoculated SARM1^KO^ mice was already significantly lower than that of the corresponding C57Bl/6 NBH inoculated control (p = 0.002, Fig 10E) and was indistinguishable from that of prion infected C57Bl/6 mice. This suggested that NRF2 may be expressed at lower levels in SARM1^KO^ mice even in the absence of prion infection. Indeed, a comparison of NRF2 expression in aged versus NBH or RML inoculated SARM1^KO^ mice showed no difference in NRF2 expression level (S2 Fig). Thus, independent of prion infection, NRF2 appears to be expressed at lower levels in SARM1^KO^ mice compared to mice expressing SARM1.

## Discussion

We have shown that prion disease is accelerated in mice in which SARM1 has been ablated. The reduced incubation times were associated with a significant increase in mitochondrial phosphorylating respiration (State 3) when compared to prion infected wild-type mice. The effect that we observed was not due simply to a lack of SARM1 since State 3 respiration in aged, uninoculated SARM1^KO^ and wild-type C57Bl/6 mice were similar. Rather, the data suggest that, in response to prion infection, SARM1 may actually suppress mitochondrial respiration.

SARM1 has been shown to play a suppressive role in mitochondrial respiration via its function as an NADase [39, 40]. Under conditions of oxidative stress, JNK-mediated phosphorylation of SARM1 activates its NADase activity, leading to increased cleavage of NAD^+^ and a drop in ATP production and mitochondrial basal and spare respiratory capacity [40]. SARM1 only acquires its NADase activity in response to brain injury or trauma [20], and it’s possible that neuronal loss or other damage induced by the accumulation of prions in the brain triggers its NADase activity leading to loss of NAD^+^ and lower rates of oxygen consumption. Consistent with this hypothesis, increased oxygen consumption has been observed following axonal injury of neurons from SARM1^KO^ mice [39]. The lower rate of phosphorylating respiration in prion infected wild-type C57Bl/6 mice when compared to SARM1^KO^ mice is also consistent with a SARM1-mediated loss of NAD^+^ in response to damage to the brain, as are our previous data demonstrating impaired mitochondrial respiration in a hamster model of prion infection [18]. Thus, SARM1-mediated regulation of mitochondrial respiration via its NADase activity may help to determine the tempo of prion disease progression. Furthermore, given that mitochondrial dysfunction is also found in other neurodegenerative protein misfolding diseases with prion-like spread, SARM1 may play a role in the progression of those diseases as well.

We also observed changes in the expression levels of proteins from mitochondrial complexes, with increased expression levels of CII and CIV and decreased expression of CV, which could impact mitochondrial respiration (Fig 9). These changes were only observed in brains from prion infected wild-type C57Bl/6 mice expressing SARM1 (Fig 9) and may be an attempt by the cell to regulate the activity of the ETC and control the production of damaging ROS during prion infection. Interestingly, the expression levels of CII, CIV and CV in SARM1^KO^ mice were already equivalent to that observed in prion infected wild-type mice (Fig 9C and 9D). Although the exact meaning of these results is unclear, they may indicate that the SARM1^KO^ mice are already predisposed to dysregulation of mitochondrial respiration which is then exacerbated by the stress of prion infection.

The most likely way in which increased mitochondrial respiration could impact prion disease is by the production of reactive oxygen species (ROS) especially via CI, where NAD^+^ is generated. Increased mitochondrial respiration is associated with higher levels of ROS which are counteracted by NRF2, a transcription factor that regulates the expression of proteins that protect against oxidative stress [30, 31]. Although a previous study reported an increase in NRF2 expression in SARM1^KO^ mice [41], we observed a decrease in NRF2 expression when compared to wild-type mice inoculated with NBH (Fig 10E), with NRF2 expression levels as low as those observed in RML prion infected C57Bl/6 mice (Fig 10). The lower expression of NRF2 that we observed is consistent with previous studies in prion infected neuroblastoma cells [36] and a genetic mouse model of prion disease [37], but inconsistent with an increase in NRF2 expression observed in CJD brain [38]. This suggests that regulation of NRF2 in response to prion infection may vary depending upon the type of prion disease. Our data also suggest that, even in the absence of prion infection, SARM1^KO^ mice may already be impaired in their response to oxidative stress, as lower levels of NRF2 could lead to a decreased ability to regulate ROS. When coupled with the higher levels of mitochondrial respiration, it’s possible that accumulation of ROS to a harmful level occurs more rapidly in SARM1^KO^ mice, contributing to a shortened disease incubation time.

The NADase activity of SARM1 is critical for axonal degeneration following nerve damage [24, 26]. The resultant reduction in NAD^+^ and ATP leads to mitochondrial depolarization and axonal degeneration [27, 28]. Loss of axons can be followed by neuronal cell death, and there have been studies suggesting that SARM1 may be involved in neuronal apoptosis following oxygen deprivation [42], mitochondrial depolarization [42], and viral infection [43]. SARM1 could therefore be involved in the axonal loss and neuronal cell death associated with prion infection. However, in agreement with an earlier study [29], we saw no difference in the amount or distribution of gray or white matter loss in SARM1^KO^ mice versus wild-type mice either midway through the disease course or at the clinical stage of disease. Thus, it does not appear that SARM1 mediated pathways of axonal and neuronal loss are the primary mechanisms involved in the loss of neurons and axons observed in the brains of RML prion infected mice.

Both prion infected C57Bl/6 and SARM1^KO^ mice had similarly reduced levels of two proteins involved in mitochondrial dynamics, MFN2 which is associated with fusion of the OMM, and Drp-1 which is required for fission of the OMM. These data are consistent with previously published data demonstrating disruptions in mitochondrial dynamics during prion infection [5, 6, 9, 38]. We also saw minimal differences between the two mouse strains in the cleavage products of OPA-1, the L- and S- forms of which are required for fusion of the IMM [33]. C57Bl/6 mice had a very small, but significant drop in L-OPA1 and a slight but insignificant increase in S-OPA1, but there was no difference between the levels of L-OPA1 and S-OP1 in SARM1^KO^ mice inoculated with NBH or prions (Fig 10). These results are consistent with previous studies which showed that, depending upon the prion strain used, OPA1 levels either decreased or remained unchanged during prion infection [5]. Overall, our data suggest that SARM1 has no detectable impact on disruptions to mitochondrial fission and fusion observed during prion infection. Thus, in terms of potential mitochondrial dysfunction, changes in the level of oxidative phosphorylation, rather than any impact on mitochondrial dynamics, appear to correlate best with the shorter disease incubation times observed in SARM1^KO^ mice.

Our data are consistent with previous work that also showed a significant decrease in disease incubation time using the same strain of SARM1^KO^ mice used in our study [29]. That study concluded lack of SARM1 led to an upregulation of the pro-apoptotic gene *Xaf1*, leading to an increase in neuronal apoptosis and shorter prion disease incubation times [29]. However, a more recent study demonstrated that overexpression of XAF1 was not due to lack of SARM1. Rather, it was due to 129/Ola genes on chromosomes 10 and 11 that were carried over during derivation of the original SARM1 knockout mouse line when it was backcrossed to C57Bl/6 mice [44]. Thus, while increased expression of XAF1 could still contribute to a more rapid disease course in prion infected mice, that effect is unlikely to be mediated by SARM1. Our data are not incompatible with an important role for increased expression of XAF1 in SARM1^KO^ mice contributing to prion disease tempo. Rather, they simply suggest another mechanism whereby the NADase function of SARM1 and its role in regulating mitochondrial respiration could also contribute to slowing prion disease progression.

Finally, we consider it unlikely that our results are due to background genes carried over during derivation of the SARM1^KO^ mice. We observed no difference in mitochondrial respiration between aged wild-type and SARM1^KO^ mice (Fig 5), indicating that genetic background differences between the C57Bl/6 and SARM1^KO^ mice were not having a constitutive effect on mitochondrial respiration. Genes for the proteins that we examined here including mitochondrial proteins from complexes CI-CV, MFN2, OPA1, Drp1, and NRF2 are not encoded on mouse chromosomes 10 and 11 and are thus not derived from the 129/Ola sequence. In addition, experiments in two other lines of SARM1^KO^ mice support a direct role for SARM1 in regulating mitochondrial respiration [44], a finding consistent with our results. While we cannot discount possible indirect effects of 129/Ola derived genes, our data are nonetheless consistent with the known role of SARM1 in response to brain injury and its activity as an NADase, as well as its ability to regulate mitochondrial biogenesis.

## Materials and methods

### Infection of mice

The animal protocol used was reviewed and approved by the Rocky Mountain Laboratories Animal Care and Use Committee (2015-004E). This study was carried out in strict accordance with the recommendations in the *Guide for the Care and Use of Laboratory Animals* of the National Institutes of Health. Animals in distress or showing symptoms of clinical prion disease were euthanized by isofluorane overdose followed by cervical dislocation.

B6.129X1-SARM1^tm1Aidi^/J mice (SARM1^KO^) and the recommended background control mouse strain C57Bl/6J mice (both from Jackson Laboratory, Bar Harbor ME) were inoculated with the mouse adapted prion strain RML-Chandler (RML). The inoculum was a 10% (w/v) brain homogenate stock suspended in 0.32M sucrose derived from the brains of clinically positive RML prion infected RML mice. RML mice are Swiss-Webster mice that have been bred and maintained in-house at the Rocky Mountain Laboratories for decades. The RML prion stock has a titer in C57Bl/10 mice of 2 x 10^8.8^ ID_50_/g. Inoculum was prepared by diluting brain homogenate 1:10 in PBS (final w/v of 1%) which was then vortexed and sonicated for 2 minutes followed by centrifugation for 1 minute at 735 x g prior to injection. Male or female mice (8–10 weeks old) were infected by injecting 50 μL of inoculum into the left hemisphere of the brain (intracerebral route) of mice anesthetized with isoflurane. The inoculum for the control group was prepared and injected exactly the same way except that a 10% (w/v) normal brain homogenate (NBH) stock from uninfected C57BL/6 mice was used. For each group, 4–12 mice were inoculated per experiment. Mice were monitored weekly and housed in HEPA-filtered cages with water and food pellets, *ad libidum*. Whole brains were collected at the clinical stage of disease as diagnosed by altered nesting and grooming habits, progressive ataxia and kyphosis. Intercurrent deaths unrelated to prion infection were not included in the final data analysis.

### Mitochondrial purification

Mitochondrial purification was performed based on the method, buffers, and reagents of Iuso et. al. [45] with the following modifications. Mouse brains were collected in 5 ml of ice-cold mitochondrial isolation buffer (MIB1 buffer: 210 nM d- Mannitol, 70 mM sucrose, 5 mM HEPES, 1 mM EGTA, and 0.5% (w/v) fatty acid-free BSA, pH 7.2 in ultrapure water) and kept on ice. Brains were cut in half along the sagittal line and the left half was immediately used to isolate mitochondria while the right half was frozen at -20°C.

For mitochondrial isolation, all procedures were done on ice. One-half of a mouse brain was cut into thirds and each piece was placed in a gentleMACS C tube (Miltenyi Biotech, Auburn, CA) containing 5 ml of ice-cold MIB1 buffer. Tissues were homogenized using the GentleMACS tissue dissociator (Miltenyi Biotech, Auburn CA) set on the Mouse Brain Mitochondria 01 program. Homogenates were centrifuged at 800 x g for 10 minutes at 4°C and supernatants were collected and pooled in a 50ml centrifugation tube. Samples were then centrifuged at 8000 x g at 4°C for 10 minutes in a fixed rotor. Following removal of the supernatant, the pellet was washed with 10ml of MIB1 buffer followed by another centrifugation at 8000 x g for 10 minutes at 4°C. The final supernatant was removed and the pellet resuspended in 0.5ml mitochondrial assay solution (MAS1: 220 nM d- Mannitol, 70 mM sucrose, 10 mM potassium dihydrogen phosphate, 5 mM magnesium chloride, 2 mM HEPES, 1 mM EGTA, and 0.2% (w/v) fatty acid-free BSA, pH 7.2 in ultrapure water).

The pellet containing mitochondria was broken up by gently pipetting up and down 15 times with a wide bore pipette and protein concentration was immediately measured using the Coomassie Plus (Bradford) Assay Kit (Thermofisher, Waltham, MA). The final protein concentration of the mitochondrial samples was 100μg/ml in MAS1 solution. The samples were supplemented with the CII substrate succinate (10mM final concentration) and the CI inhibitor rotenone (2 μM final concentration) and used in the mitochondrial coupling assay.

### Seahorse XFe96 mitochondrial coupling assay

Mitochondrial preparations were added to Seahorse 96eXF assay plates that had been coated with poly-D-lysine. Immediately prior to performing the bioenergetics assay, mitochondria were centrifuged at 4000 x g at 4°C for 10 minutes to form a monolayer on the bottom of the poly-d-lysine coated Seahorse plate. The optimal protein concentration per well of isolated mitochondria as well as the optimal concentrations of ADP, oligomycin, carbonyl cyanide p-trifluoromethoxyphenylhydrazone (FCCP), and antimycin A were systematically determined by testing different dilutions in mitochondrial coupling assays using the automated Seahorse XF96 Analyzer (Agilent) and a 96 well plate format. The optimized final concentrations of the assay components were determined to be 5μg/50μl of mitochondria, 1mM ADP (saturating substrate), 1μM oligomycin (CV inhibitor), 2μM FCCP (uncoupling reagent) and 4μM antimycin A (CIII inhibitor). These concentrations resulted in reproducible oxygen consumption rates (OCR) and respiratory control ratio (RCR) values consistent with those previously reported for healthy brain mitochondria [32].

Mice infected with RML prions were assayed on the same plate and under the same conditions as control mice inoculated with NBH. Only assays with a minimum of 12 replicate wells of usable data per mouse were used for data analysis [46]. For studies on aged SARM1^KO^ and C57Bl/6J, mice aged 288, 289, or 324 days were assayed with one SARM1^KO^ and one C57Bl/6J mouse per 96 well plate. The CII substrate succinate and the CI inhibitor rotenone were present throughout the assay. Parameters for the assay were set to a 30 second mixing phase followed by a 10 second rest period and 2 minute read period for each measurement. This was repeated twice for basal readings and then twice after each injection. After an equilibration period of 14–16 minutes, basal readings for State 2 were taken after which a saturating concentration of 1mM ADP was injected to determine the OCR for mitochondrial respiration. At 10 minutes, 1μM of the CV inhibitor oligomycin was added to determine the amount of oxygen consumption in the absence of phosphorylation (i.e. proton leak) followed by the addition of 2μM FCCP at 16 minutes to fully uncouple respiration from phosphorylation and determine the oxidative capacity of CII through CIV. Finally, in order to determine the level of non-mitochondrial respiration, 4μM of antimycin A was injected at 22 minutes to inhibit CIII and block the electron transport chain (ETC). To correct for background, wells on each corner of the plate contained 50 μl of MAS1 solution plus 10 mM succinate and 2 μM rotenone.

### Calculation of OCR and RCR

The OCR for each state as well as the RCR of all samples were determined as described in Iuso et. al. Briefly, OCRs for States 2, 3, 4o and 3u were calculated by subtracting the average non-mitochondrial respiration from two data points from the average OCR of the two datapoints taken for each state. To determine the RCR for the coupled state, State 3 was divided by State 4o. The RCR of the uncoupled state (RCR 3u) was calculated by dividing State 3u by State 4o. Statistics were calculated using GraphPad Prism (v8.0.2.263).

### SDS-PAGE and western blotting

Processing of brains from RML and NBH inoculated mice for the detection of PrP^Sc^ as well as SDS-PAGE, transfer of proteins to polyvinylidene difluoride (PVDF) membrane (Millipore, Burlington MA), and membrane development were all done as described previously [47]. For detection of proteins other than PrP^Sc^, digestion with proteinase K was omitted. For all samples, 0.12 mg brain equivalents were loaded per well.

The primary antibody used to detect PrP^Sc^ was the anti-PrP mouse monoclonal antibody 6D11 (PrP residues 95–105, Biolegend, San Diego CA) at a dilution of 1:5,000. For detection of subunit proteins from mitochondrial complexes CI-CV, the Total OXPHOS Rodent WB Antibody Cocktail (Abcam, Waltham MA) was used at a dilution of 1:500. This antibody mixture recognizes key proteins in each of the complexes of the ETC: NADH:ubiquinone oxidoreductase subunit B8 (CI), succinate dehydrogenase [ubiquinone] iron-sulfur subunit (CII), cytochrome b-c1 complex subunit 2 (CIII), cytochrome c oxidase subunit I (CIV), and the alpha subunit of CV. As a positive control for identification of mitochondrial proteins, 2.5–5μl of a 1.5mg/ml mitochondrial extract from rat heart tissue (Abcam, Waltham MA) was loaded per well.

The protein NRF2 was detected using an anti-NRF2 mouse monoclonal antibody (Novus Biologicals, Littleton CO) at a dilution of 1:500. Mitofusion 2 (MFN2) was detected using a rabbit monoclonal antibody at 1:2000, optical opacity protein (OPA1) was detected using a rabbit monoclonal antibody diluted 1:1000, dynamin related protein (Drp1) was detected using a rabbit monoclonal antibody diluted 1:1000, and β-actin was detected using a rabbit monoclonal antibody diluted 1:10,000. All of these antibodies were purchased from Cell Signaling Technology, Danvers MA. All rabbit primary antibodies were detected with a 1:250,000 dilution of a donkey anti-rabbit secondary antibody conjugated to horse radish peroxidase (GE Healthcare, Chicago IL). A sheep anti-mouse antibody conjugated to horse radish peroxidase (GE Healthcare, Chicago IL) was used at a dilution of 1:250,000 to detect 6D11, NRF2, and the mitochondrial protein antibody cocktail. Following an incubation in secondary antibody for one hour at room temperature, all blots were developed using the SuperSignal West Femto Maximum Sensitivity Substrate (ThermoFisher Scientific, Waltham MA) for detection on X-ray film.

### Immunohistochemistry

For all mouse strains, 4 mice were analyzed for each timepoint and condition. Formalin fixed tissue was processed and embedded in paraffin. Sections (5 microns) were cut using a standard Leica microtome, placed on positively charged glass slides, and air-dried overnight at room temperature. Deparaffinization, hydration and staining of tissue sections were performed on the automated Discovery XTstaining system (Ventana Medical Systems, Oro Valley AZ). Antigen retrieval was done using the extended CC1 protocol (cell conditioning buffer containing Tris-Borate-EDTA, pH 8.0, for approximately 44 minutes at 100°C). Following antigen retrieval, slides were incubated for 60 minutes at 37°C with the anti-PrP rabbit monoclonal antibody EP1802Y (GeneTex, Irvine CA), which recognizes C-terminal PrP amino acids 217–226, at a dilution of 1:6,000 in antibody dilution buffer (Ventana ADB250). The secondary antibody was biotinylated goat anti-rabbit IgG Ready-to-use Super Sensitive Rabbit (Bio-genex, Fremont CA) which was applied for 32 min at 37°C. Slides were developed using the DABMap chromagen detection kit and Discovery S Block RUO (Ventana Medical Systems, Oro Valley AZ) followed by hematoxylin counterstain.

Following deparaffinization and hydration, hematoxylin and eosin (H&E) staining was also performed. Slides were incubated in hematoxylin (ThermoFisher Scientific, Waltham MA) for 20 minutes according to the manufacturer’s instructions. To optimize the contrast and definition between hematoxylin (purple/blue) and eosin (pink) stained tissue, hematoxylin stained tissue was rinsed in tap water, immersed in 0.75% ammonium hydroxide in 70% alcohol, and rinsed with water followed by immersion in Surgipath Blue Buffer 8 (ThermoFisher Scientific, Waltham MA). Following a final water rinse, slides were incubated for 4 minutes in Eosin (Shandon Eosin Y alcoholic) and coverslipped.

### Brain lesion profile

Spongiform change in the brains of RML prion infected mice at the clinical stage of infection was assessed using sagittal slide sections stained with hematoxylin and eosin under a 10X objective. For three mice from each strain, nine brain regions were analyzed: frontal cortex, thalamus, hypothalamus, hippocampus, superior colliculus, midbrain, pons, medulla, and cerebellum. The extent of vacuolation was scored as follows: 0, no vacuoles; 1, scarce and unevenly scattered vacuoles that are widely distributed; 2, multiple evenly scattered vacuoles; 3, moderate number of evenly scattered vacuoles; 4, many vacuoles with some confluence; 5, dense vacuolation with a lacy appearance.

### Quantitation of protein expression

The UN-SCAN-IT gel program (v7.1) was used to quantify proteins detected by western blot. Proteins were quantified by summing the pixel intensities in user defined rectangles encompassing the band of interest. Identically sized rectangles were used to quantify the background intensity which was then subtracted from the pixel sums for each quantified band. Protein levels were normalized to mouse β-actin by dividing background corrected pixel sums for the protein of interest by background corrected pixel sums from β-actin bands in the same lanes. The individual ratios of each lane were averaged together and statistically analyzed using GraphPad Prism (v8.0.2.263).

## Supporting information

S1 Fig Similar levels of PrP^Sc^ and spongiform change in RML prion infected SARM1^KO^ and C57Bl/6 mice. (A) Quantitation of PrP^Sc^ in brain homogenate from clinically positive RML infected C57Bl/6 (black bar) and SARM1^KO^ (white bar) mice. Data are derived from the western blot data shown in Fig 3. Mean ± S.E.M. is shown for 5 mice. a.u. = arbitrary units. Statistical analysis using the unpaired Student’s t-test with Welch’s correction showed no significant difference between the two datasets. (B) Brain lesion profile of clinically positive RML infected C57Bl/6 (closed circles) and SARM1^KO^ (open circles) mice. Mean ± S.E.M. is shown for 3 mice. Statistical analysis using the unpaired Student’s t-test with Welch’s correction showed no significant differences between the two datasets. FC = frontal cortex; Hypothal = hypothalamus; HC = hippocampus; SC = superior colliculus; Cb = cerebellum.
(PDF)

S2 Fig NRF2 expression is unaltered in prion infected SARM1^KO^ mice. Expression of NRF2 in aged (gray bar), NBH inoculated (black bar), or prion infected SARM1^KO^ mice (white bar). Results were obtained by immunoblot analysis of mouse brain homogenate developed with an anti-NRF2 mouse monoclonal antibody at a dilution of 1:500. Data were normalized to mouse actin (Relative Intensity) and were calculated from n = 5 animals for each condition. Statistical analysis using a 1-way ANOVA with Dunnett’s post-test and aged SARM1^KO^ mice as the control, showed no statistical difference in NRF2 expression between the samples. Mean ± SEM is shown.
(PDF)

S1 Table Oxygen consumption rates and respiratory control ratios in response to the CII substrate succinate in mitochondria from aged C57BL/6 and SARM1^KO^ mice. (PDF)

S2 Table Oxygen consumption rates and respiratory control ratios in response to the CII substrate succinate in mitochondria from C57BL/6 mice inoculated with RML prions or NBH. (PDF)

S3 Table Oxygen consumption rates and respiratory control ratios in response to the CII substrate succinate in mitochondria from SARM1^KO^ mice inoculated with RML prions or NBH. (PDF)

S1 Raw images (PDF)
