# Supplementary figures and images for: Lack of the immune adaptor molecule SARM1 accelerates disease in prion infected mice and is associated with increased mitochondrial respiration and decreased expression of NRF2
Source: PLoS One. 2022 May 4;17(5):e0267720. doi: 10.1371/journal.pone.0267720 (PMC9067904; doi:10.1371/journal.pone.0267720)

S1 Figure

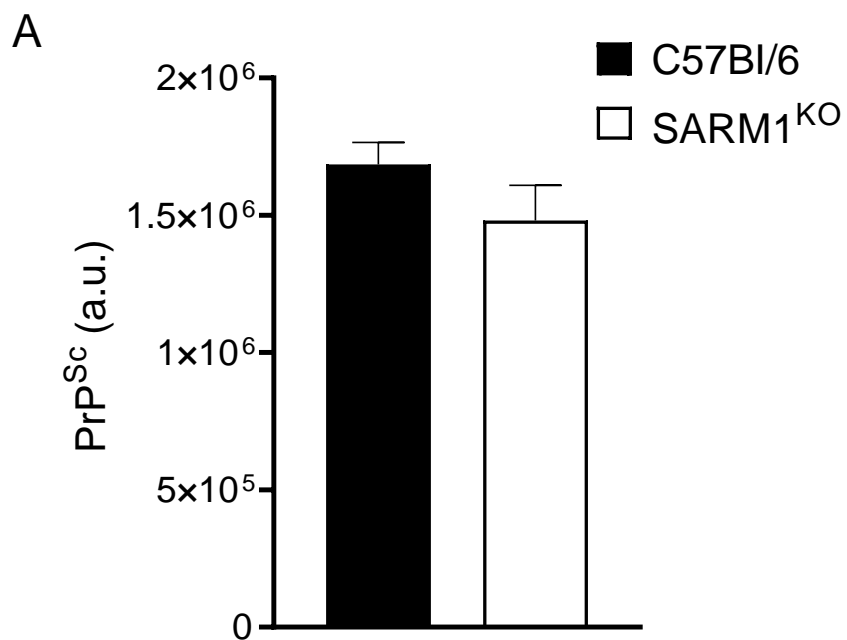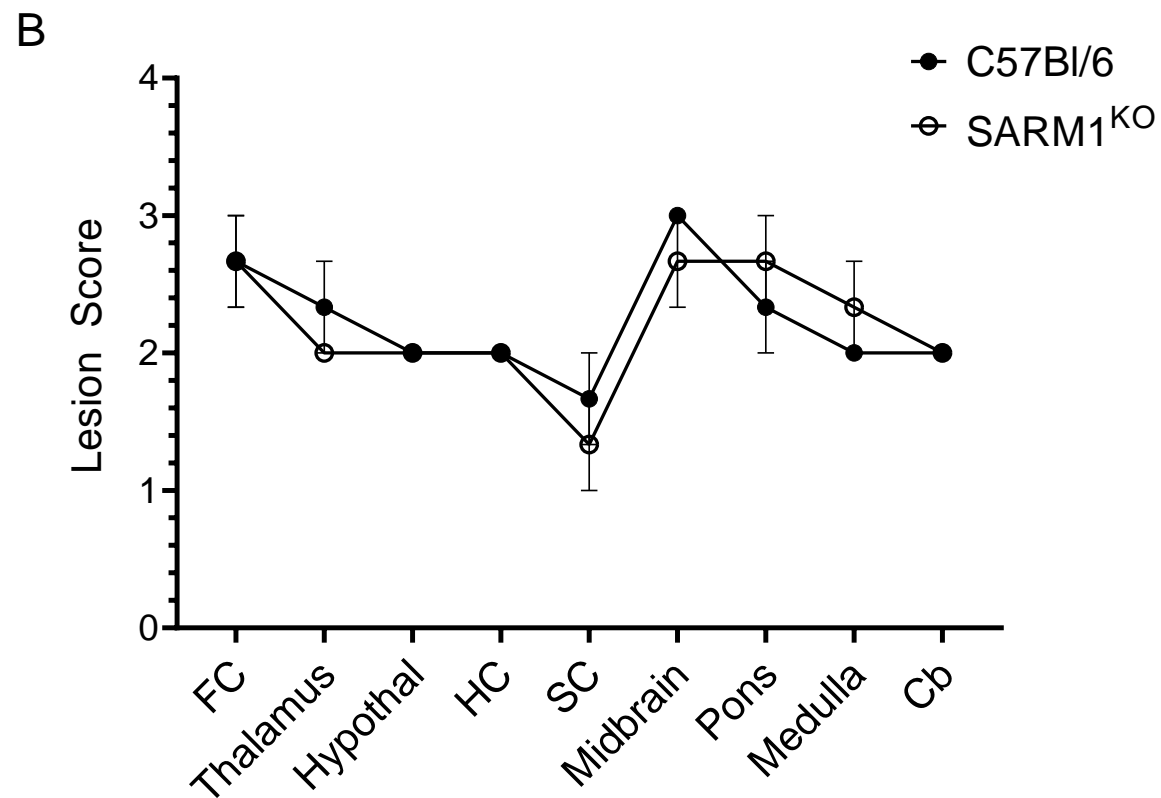

Supplement: S1 Fig — (A) Quantitation of PrPSc in brain homogenate from clinically positive RML infected C57Bl/6 (black bar) and SARM1KO (white bar) mice. Data are derived from the western blot data shown in Fig 3. Mean ± S.E.M. is shown for 5 mice. a.u. = arbitrary units. Statistical analysis using the unpaired Student’s t-test with Welch’s correction showed no significant difference between the two datasets. (B) Brain lesion profile of clinically positive RML infected C57Bl/6 (closed circles) and SARM1KO (open circles) mice. Mean ± S.E.M. is shown for 3 mice. Statistical analysis using the unpaired Student’s t-test with Welch’s correction showed no significant differences between the two datasets. FC = frontal cortex; Hypothal = hypothalamus; HC = hippocampus; SC = superior colliculus; Cb = cerebellum. (PDF) [file pone.0267720.s001.pdf]

S2 Figure

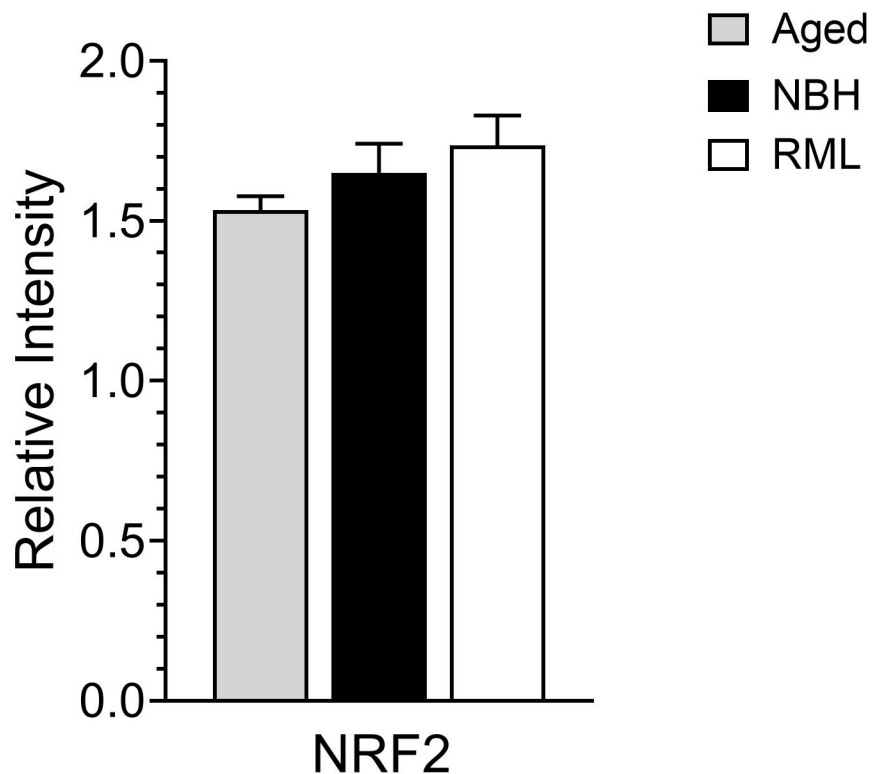

Supplement: S2 Fig — Expression of NRF2 in aged (gray bar), NBH inoculated (black bar), or prion infected SARM1KO mice (white bar). Results were obtained by immunoblot analysis of mouse brain homogenate developed with an anti-NRF2 mouse monoclonal antibody at a dilution of 1:500. Data were normalized to mouse actin (Relative Intensity) and were calculated from n = 5 animals for each condition. Statistical analysis using a 1-way ANOVA with Dunnett’s post-test and aged SARM1KO mice as the control, showed no statistical difference in NRF2 expression between the samples. Mean ± SEM is shown. (PDF) [file pone.0267720.s002.pdf]
