# Supplementary material for: Lack of the immune adaptor molecule SARM1 accelerates disease in prion infected mice and is associated with increased mitochondrial respiration and decreased expression of NRF2
Source: PLoS One. 2022 May 4;17(5):e0267720. doi: 10.1371/journal.pone.0267720 (PMC9067904; doi:10.1371/journal.pone.0267720)
Supplement: S1 Table — (PDF) [file pone.0267720.s003.pdf]

S1 Table. Oxygen consumption rates and respiratory control ratios in response to the CII substrate succinate in mitochondria from aged C57BL/6 and SARM1^{KO} mice.

		OCR[a]		
		Pair 1[b]	Pair 2	Pair 3
State 2	C57Bl/6	248 ± 4	271 ± 5	178 ± 5
	SARM1^{KO}	260 ± 7	233 ± 3	198 ± 4
	p-value[c]	NS	<0.0001	0.004
State 3	C57Bl/6	434 ± 19	510 ± 19	386 ± 15
	SARM1^{KO}	451 ± 22	453 ± 6	437 ± 9
	p-value	NS	0.009	0.006
State 4o	C57Bl/6	147 ± 10	191 ± 23	97 ± 3
	SARM1^{KO}	194 ± 20	146 ± 12	101 ± 4
	p-value	0.04	NS	NS
State 3u	C57Bl/6	326 ± 21	399 ± 23	280 ± 25
	SARM1^{KO}	367 ± 24	350 ± 10	335 ± 20
	p-value	NS	NS	NS
Non-mito[d]	C57Bl/6	22 ± 2	23 ± 3	20 ± 2
	SARM1^{KO}	24 ± 2	24 ± 1	20 ± 3
	p-value	NS	NS	NS
		RCR		
RCR 3/4o	C57Bl/6	3.1 ± 0.2	3.2 ± 0.3	4.0 ± 1.0
	SARM1^{KO}	2.6 ± 0.2	3.5 ± 0.2	4.5 ± 0.2
	p-value	NS	NS	NS
RCR 3u/4o	C57Bl/6	2.4 ± 0.2	2.4 ± 0.2	2.9 ± 0.2
	SARM1^{KO}	2.1 ± 0.2	2.7 ± 0.1	3.4 ± 0.2
	p-value	NS	NS	NS

[a] OCR = mean ± SEM oxygen consumption rate in pmol/min.

[b] each mouse pair represents a single assay done in one 96 well plate. Number of replicate wells: Pair 1, C57Bl/6=18, SARM1^{KO}=24; Pair 2, C57Bl/6=21, SARM1^{KO}=31; Pair 3, C57Bl/6=19, SARM1^{KO}=29.

[c]unpaired t-test with Welch's correction comparing C57BL/6 and SARM1^{KO} samples. NS = Not Significant.

[d]non-mitochondrial respiration.
