# Supplementary material for: Lack of the immune adaptor molecule SARM1 accelerates disease in prion infected mice and is associated with increased mitochondrial respiration and decreased expression of NRF2
Source: PLoS One. 2022 May 4;17(5):e0267720. doi: 10.1371/journal.pone.0267720 (PMC9067904; doi:10.1371/journal.pone.0267720)
Supplement: S2 Table — (PDF) [file pone.0267720.s004.pdf]

S2 Table. Oxygen consumption rates and respiratory control ratios in response to the CII substrate succinate in mitochondria from C57BL/6 mice inoculated with RML prions or NBH.

		OCR[a]				
		Pair 1[b]	Pair 2	Pair 3	Pair 4	Pair 5
State 2	NBH	98 ± 48	180 + 6	129 ± 9	169 ± 11	139 ± 12
	RML	101 ± 3	125 ± 4	151 ± 8	127 ± 8	174 ± 10
	p-value[c]	NS	<0.0001	0.07	0.003	0.04
State 3	NBH	292 ± 10	590 ± 14	300 ± 16	330 ± 11	272 ± 11
	RML	305 ± 8	393 ± 11	337 ± 6	264 ± 10	374 ± 6
	p-value	NS	<0.0001	0.04	<0.0001	<0.0001
State 4o	NBH	96 ± 14	195 ± 20	73 ± 7	97 ± 6	69 ± 7
	RML	104 ± 10	142 ± 19	70 ± 4	69 ± 7	98 ± 6
	p-value	NS	NS	NS	0.003	0.005
State 3u	NBH	237 ± 11	522 ± 29	209 ± 18	204 ± 10	161 ± 13
	RML	266 ± 9	342 ± 13	204 ± 12	151 ± 12	224 ± 11
	p-value	0.05	<0.0001	NS	0.002	0.0008
Non-mito[d]	NBH	17 ± 3	42 ± 1	19 ±1	15 ± 4	12 ± 3
	RML	14 ± 2	27 ± 1	22 ± 5	14 ± 5	12 ± 3
	p-value	NS	<0.0001	NS	NS	NS
		RCR				
RCR 3/4o	NBH	3.6 ± 0.3	3.5 ± 0.3	4.3 ± 0.3	3.6 ± 0.2	4.2 ± 0.3
	RML	3.4 ± 0.3	3.4 ± 0.3	5.1 ± 0.2	4.9 ± 0.7	4.2 ± 0.3
	p-value	NS	NS	0.02	NS	NS
RCR 3u/4o	NBH	2.9 ± 0.2	3.1 ± 0.3	3.0 ± 0.3	2.2 ± 0.1	2.5 ± 0.2
	RML	3.0 ± 0.3	2.9 ± 0.2	3.0 ± 0.2	2.5 ± 0.2	2.5 ± 0.2
	p-value	NS	NS	NS	NS	NS

[a] OCR = mean ± SEM oxygen consumption rate in pmol/min.

[b] each mouse pair represents a single assay done in one 96 well plate. Number of replicate wells: Pair 1, NBH=16, RML=23; Pair 2, NBH=20, RML=20; Pair 3, NBH=14, RML=29; Pair 4, NBH=22, RML=17; Pair 5, NBH=12, RML=22.

[c]unpaired t-test with Welch's correction comparing NBH and RML inoculated samples for a given pair. NS = Not Significant.

[d]non-mitochondrial respiration.
