# Supplementary material for: Lack of the immune adaptor molecule SARM1 accelerates disease in prion infected mice and is associated with increased mitochondrial respiration and decreased expression of NRF2
Source: PLoS One. 2022 May 4;17(5):e0267720. doi: 10.1371/journal.pone.0267720 (PMC9067904; doi:10.1371/journal.pone.0267720)
Supplement: S3 Table — (PDF) [file pone.0267720.s005.pdf]

S3 Table. Oxygen consumption rates and respiratory control ratios in response to the CII substrate succinate in mitochondria from SARM1^{KO} mice inoculated with RML prions or NBH.

		OCR[a]					
		Pair 1[b]	Pair 2	Pair 3	Pair 4	Pair 5	Pair 6
State 2	NBH	131 ± 5	147 ± 4	159 ± 5	151 ± 3	99 ± 3	189 ± 4
	RML	213 ± 4	183 ± 4	166 ± 6	181 ± 3	118 ± 5	153 ± 6
	p-value[c]	<0.0001	<0.0001	NS	<0.0001	0.001	<0.0001
State 3	NBH	527 ± 10	421 ± 8	402 + 14	437 ± 7	311 ± 8	450 ± 9
	RML	769 ± 17	484 ± 10	448 ± 9	527 ± 8	351 ± 8	381 ± 10
	p-value	<0.0001	<0.0001	0.011	<0.0001	0.0007	<0.0001
State 4o	NBH	185 ± 17	133 ± 13	127 ± 9	139 ± 12	89 ± 8	135 ± 12
	RML	342 ± 42	134 ± 9	113 ± 5	149 ± 9	91 ± 8	86 ± 7
	p-value	0.003	NS	NS	NS	NS	0.001
State 3u	NBH	435 ± 23	337 ± 19	352 ± 21	387 ± 13	200 ± 13	271 ± 14
	RML	623 ± 20	408 ± 16	368 ± 18	462 ± 12	225 ± 12	243 ± 17
	p-value	<0.0001	0.007	NS	0.0002	NS.	NS
Non-mito[d]	NBH	43 ± 2	25 ± 1	28 ± 2	32 ± 2	25 ± 2	16 ± 2
	RML	53 ± 3	26 ± 1	30 ± 2	36 ± 1	21 ± 3	23 ± 5
	p-value	0.003	NS	NS	NS	NS	NS
		RCR					
RCR 3/4o	NBH	3.2 ± 0.3	3.7 ± 0.2	3.4 ± 0.2	3.5 ± 0.3	3.9 ± 0.3	3.7 ± 0.2
	RML	2.8 ± 0.4	3.9 ± 0.2	4.1 ± 0.1	3.7 ± 0.2	4.6 ± 0.4	4.8 ± 0.4
	p-value	NS	NS	0.02	NS	NS	0.03
RCR 3u/4o	NBH	2.6 ± 0.2	2.8 ± 0.2	3.0 ± 0.3	3.1 ± 0.2	2.5 ± 0.2	2.2 ± 0.2
	RML	2.4 ± 0.3	3.3 ± 0.2	3.4 ± 0.2	3.3 ± 0.2	2.9 ± 0.3	2.9 ± 0.2
	p-value	NS	NS	0.02	NS	NS	0.02

[a] OCR = mean ± SEM for oxygen consumption rate in pmol/min.

[b] each mouse pair represents a single assay done in one 96 well plate. Number of replicate wells: Pair 1, NBH=16, RML=15; Pair 2, NBH=28, RML=23; Pair 3, NBH=17, RML=19; Pair 4, NBH=16, RML=22; Pair 5, NBH=19, RML=28; Pair 6, NBH=23, RML=14.

[c]Unpaired t-test with Welch's correction comparing NBH and RML samples for a given pair. NS = Not Significant.

[d]non-mitochondrial respiration.
