# Supplementary material for: Lack of the immune adaptor molecule SARM1 accelerates disease in prion infected mice and is associated with increased mitochondrial respiration and decreased expression of NRF2
Source: PLoS One. 2022 May 4;17(5):e0267720. doi: 10.1371/journal.pone.0267720 (PMC9067904; doi:10.1371/journal.pone.0267720)
Supplement: S1 Raw images — (PDF) [file pone.0267720.s006.pdf]

Figure 3: Raw Data Blots

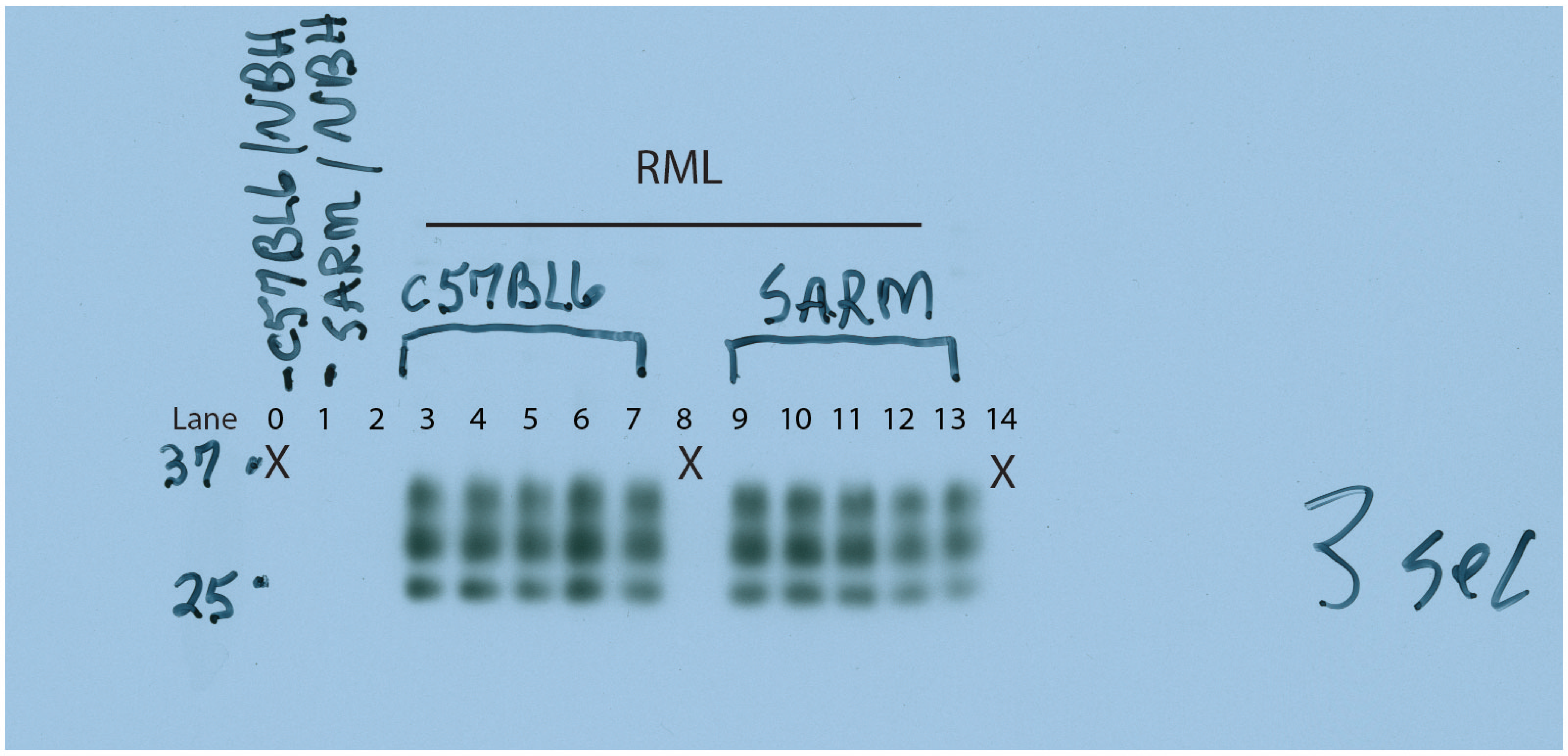

Image captured using Epson Expression 10000 XL
Image converted to gray scale for final publication

Figure 9: Raw Data Blots

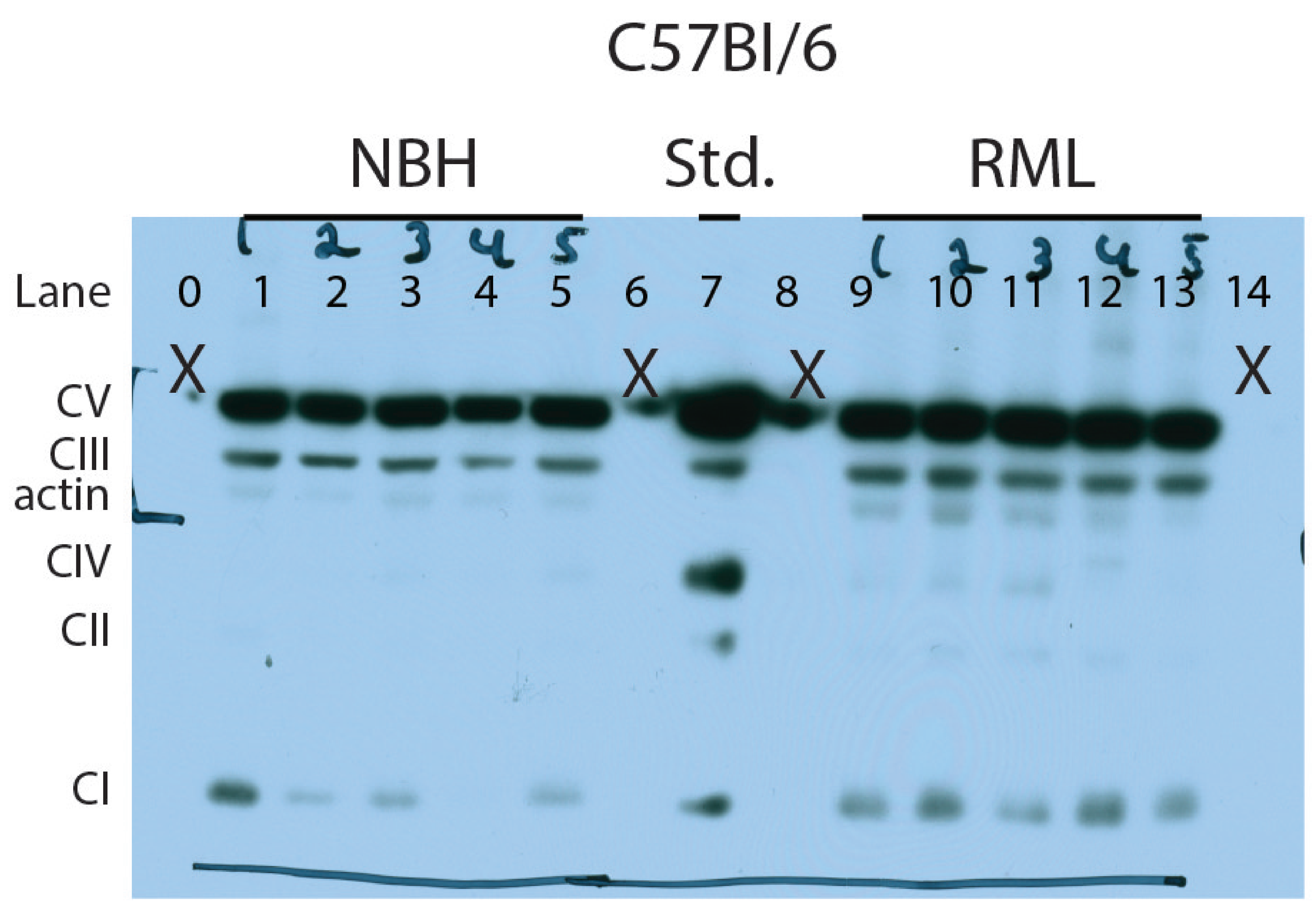

Panel A - Upper Left / Right 6 sec

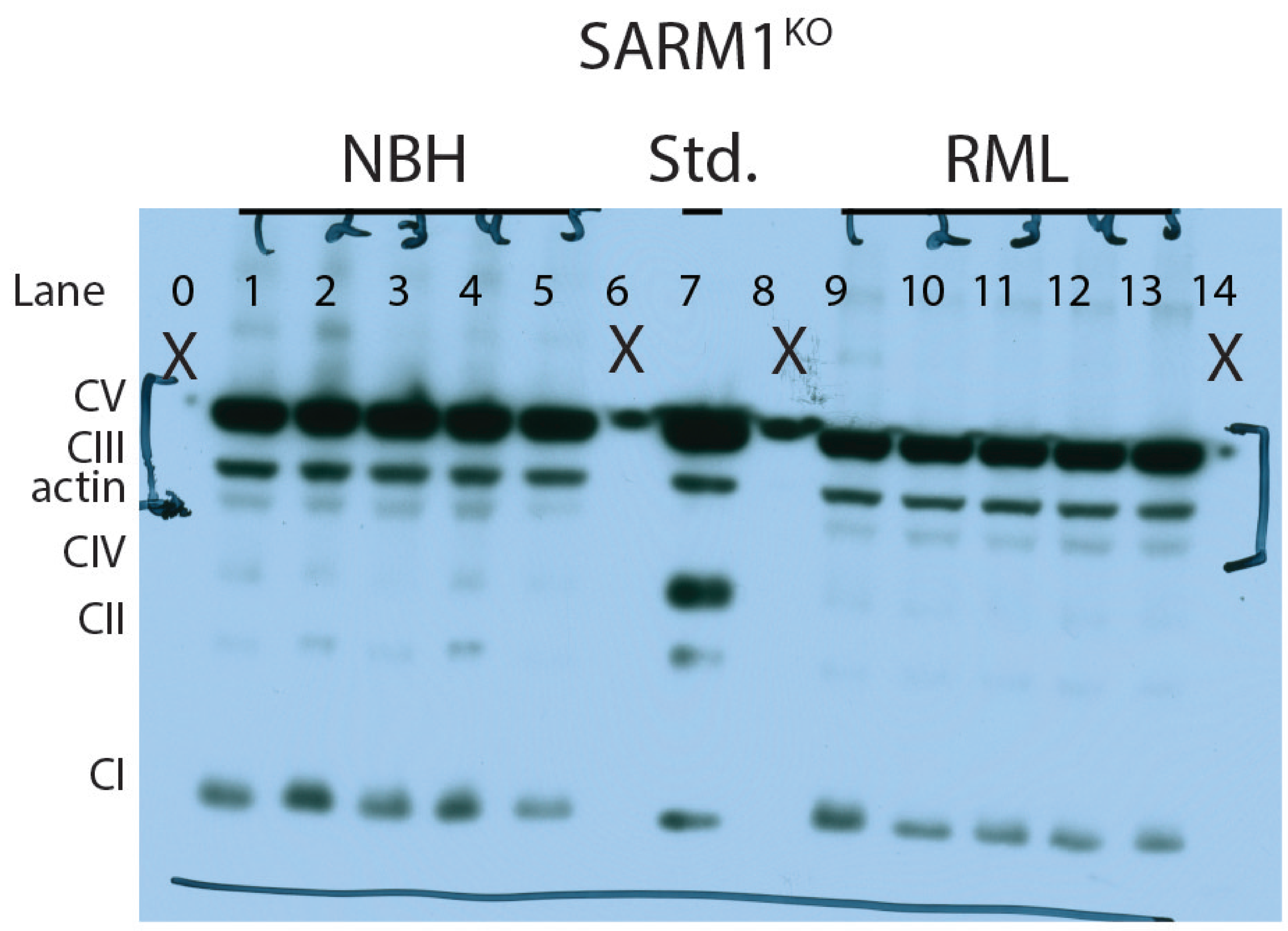

Panel B - Upper Left / Right 6 sec

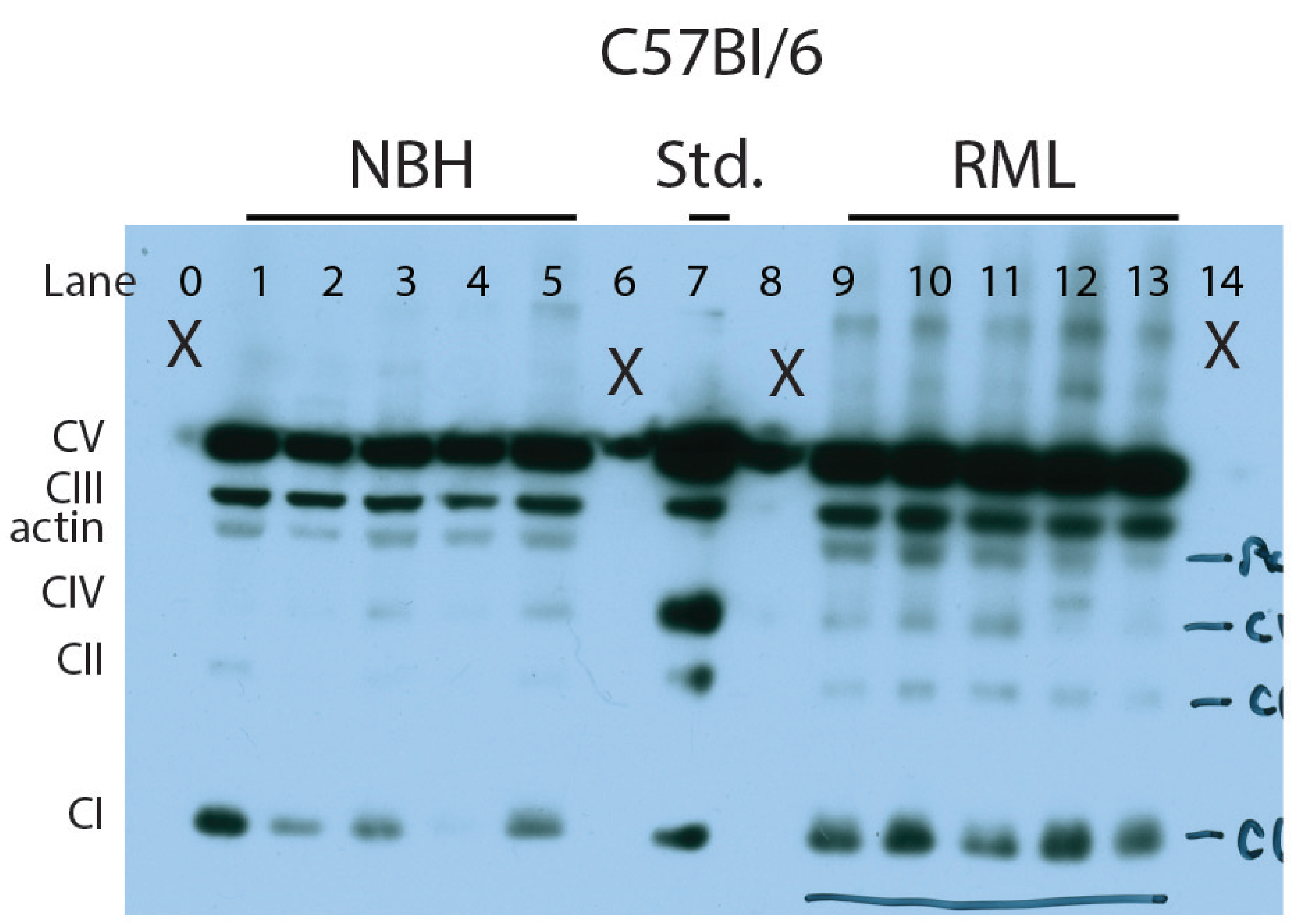

Panel A - Lower Right
10 sec

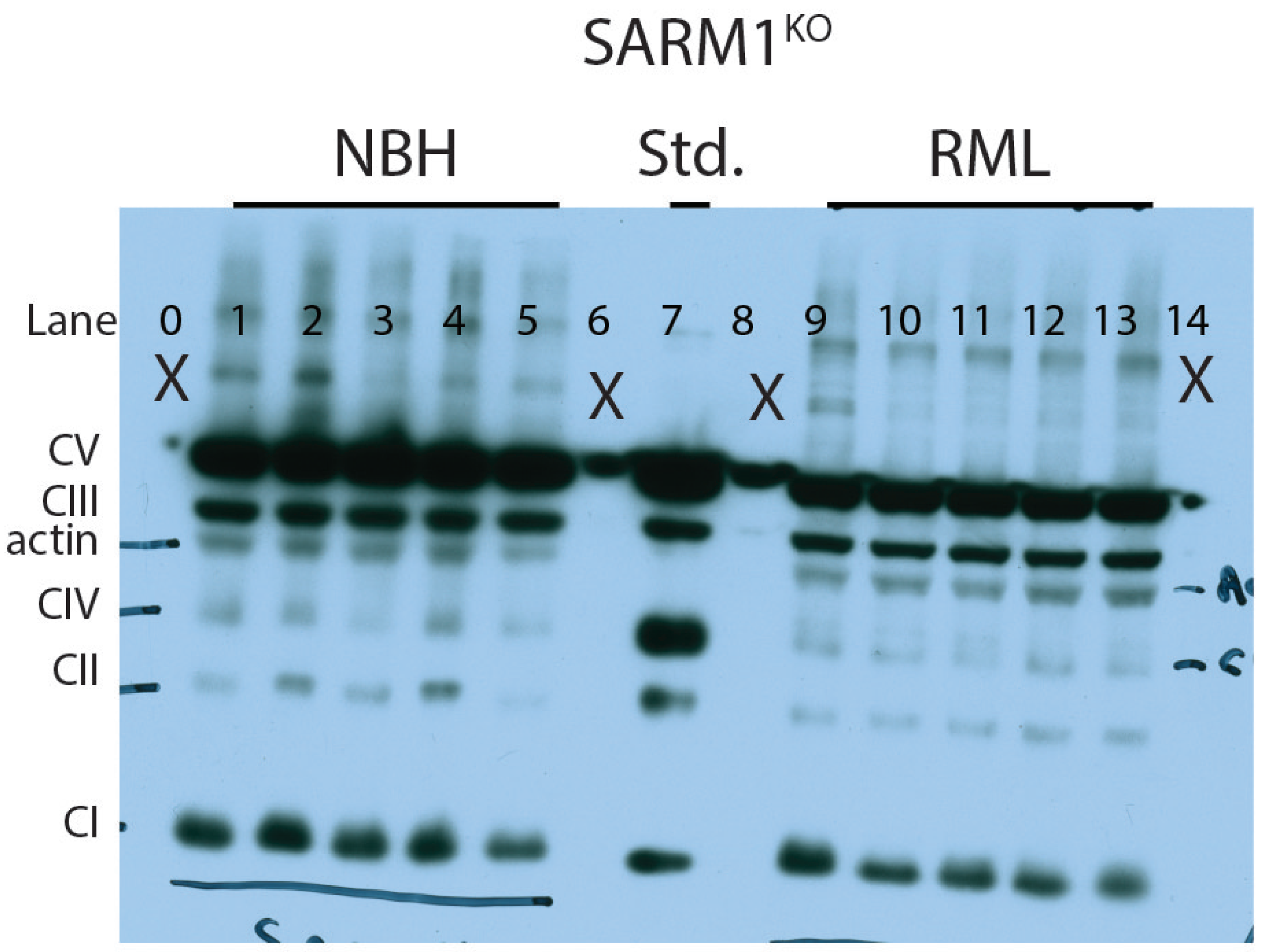

Panel B - Lower Left / Right 10 sec

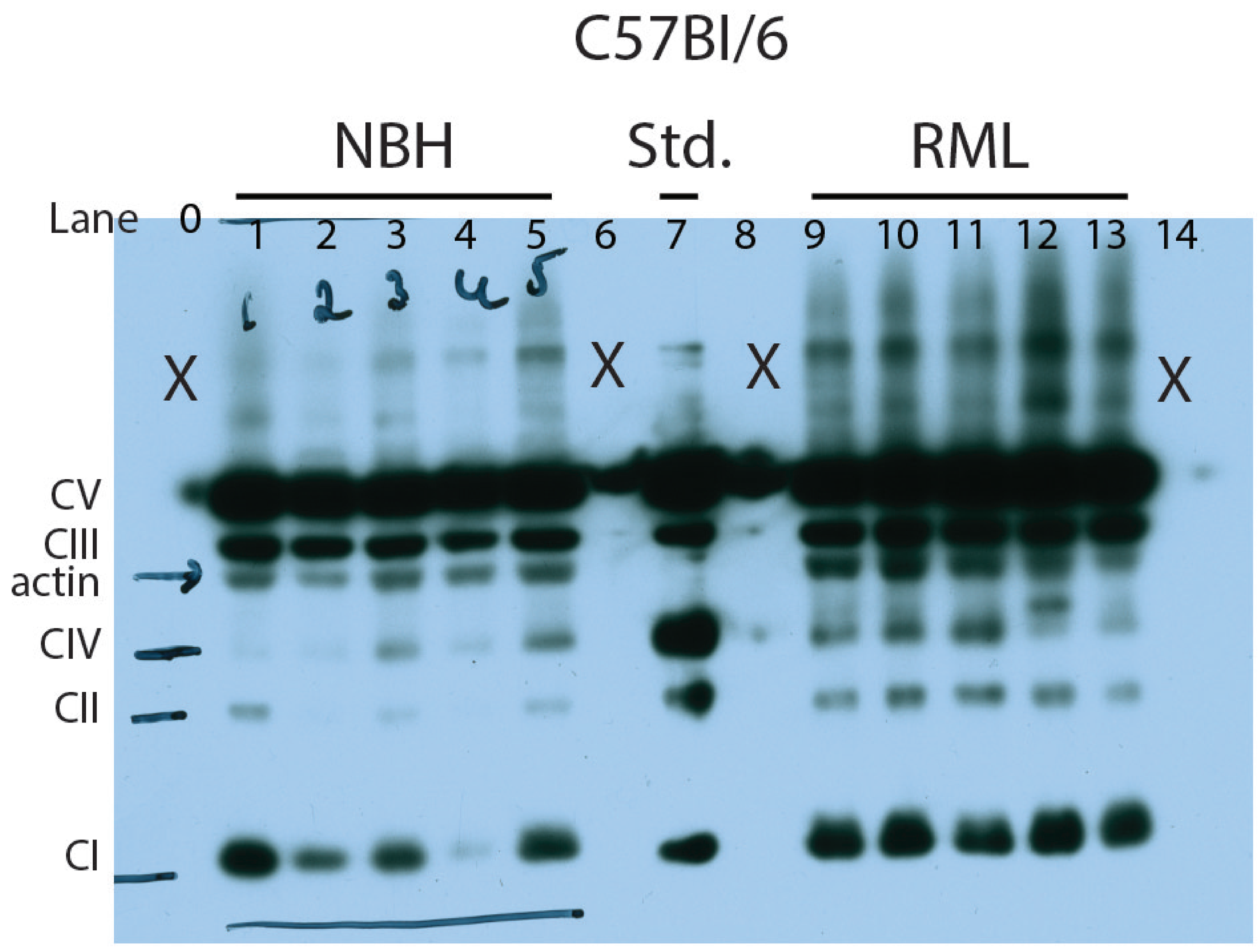

Panel A - Lower Left
30 sec

Image captured using Epson Expression 10000 XL
Image converted to gray scale for final publication

Std. = Standard, mitochondrial extract from rat heart tissue (Abcam, Waltham MA)

Figure 10: Raw Data Blots - (MFN2, Drp1)

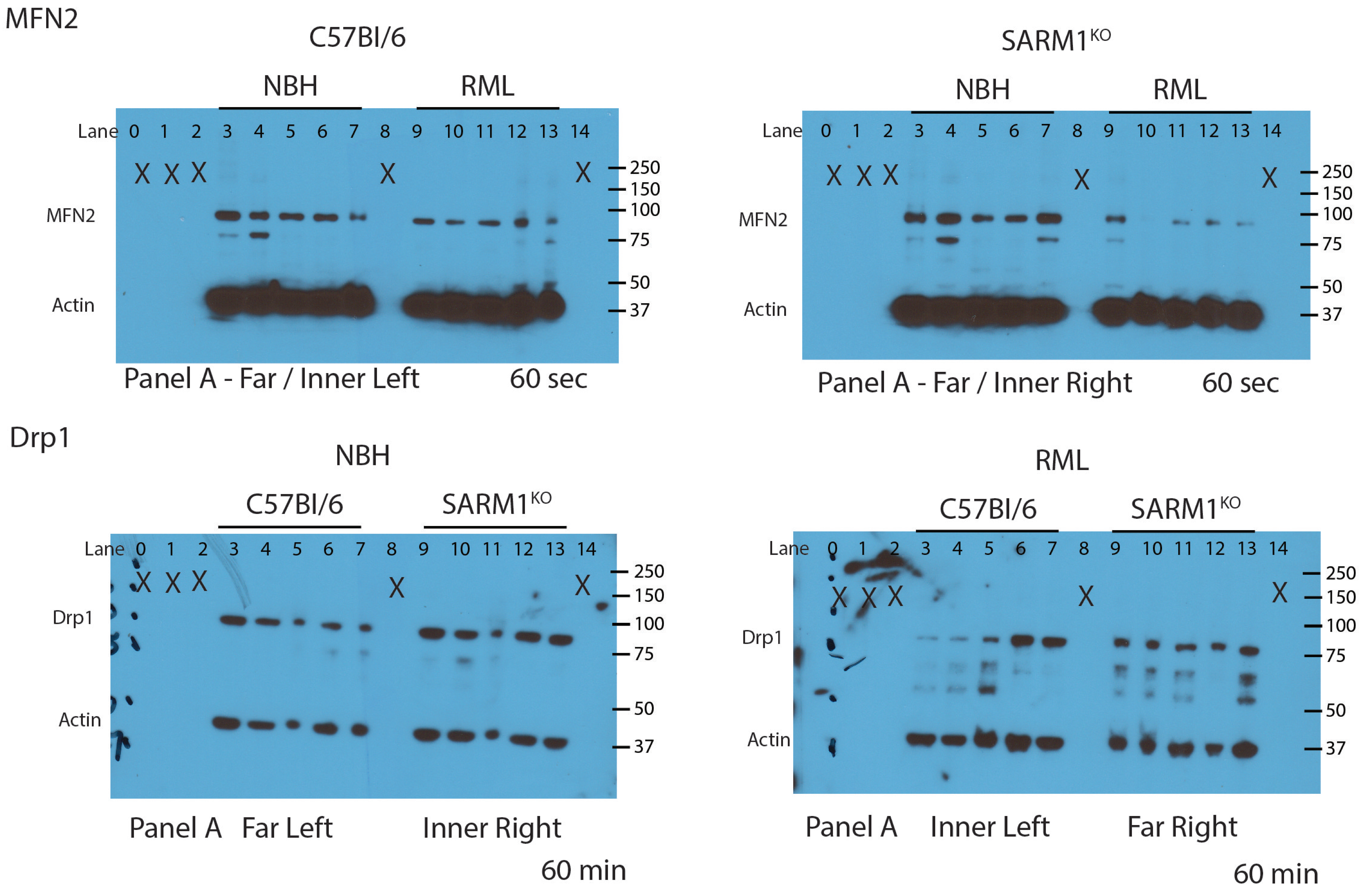

Image captured using Epson Expression 10000 XL
Image converted to gray scale for final publication

Figure 10: Raw Data Blots - (OPA1, NRF2, Actin)

OPA1 L/S

NBH

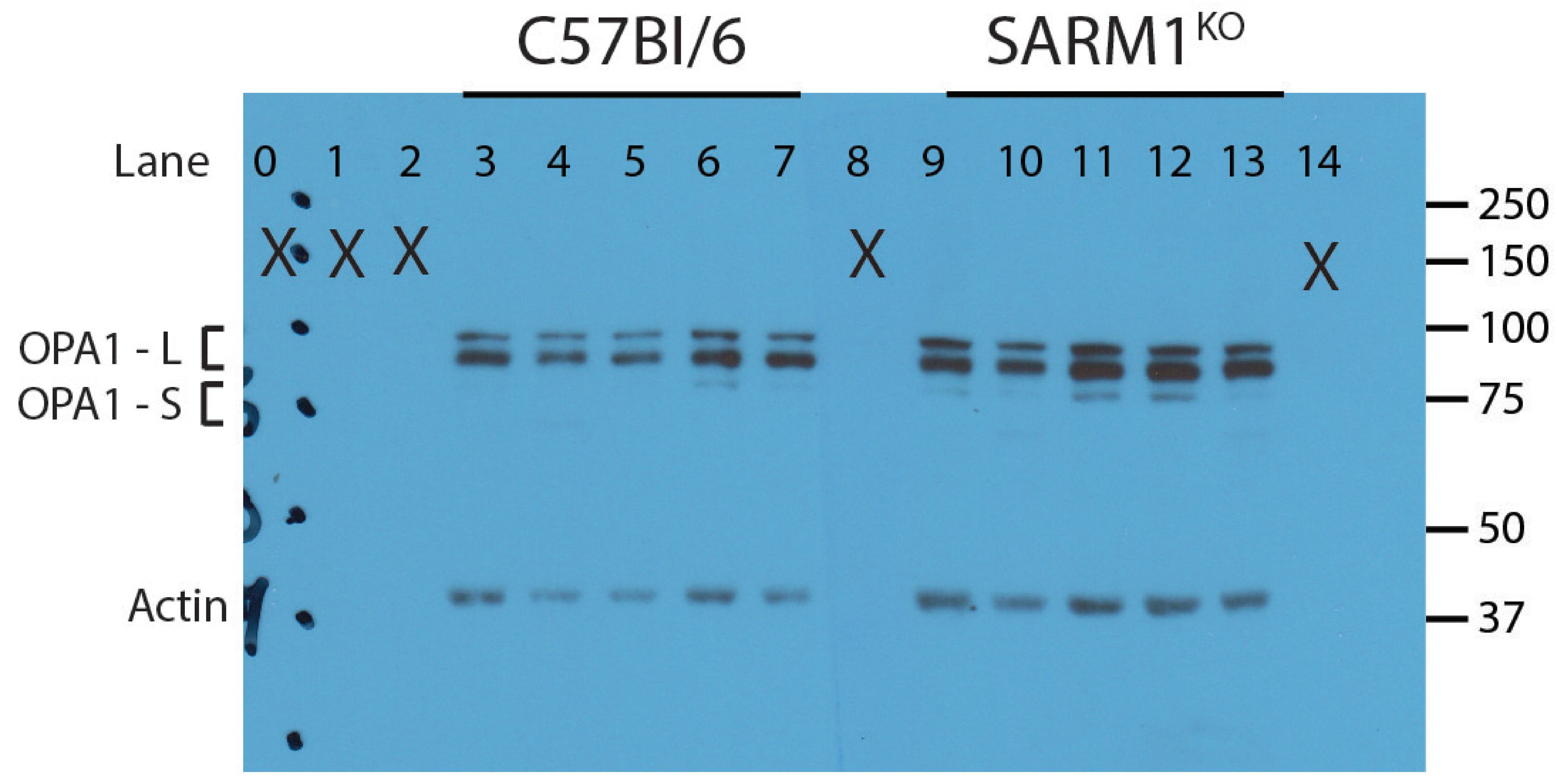

Panel A Far Left Inner Right

60 sec

RML

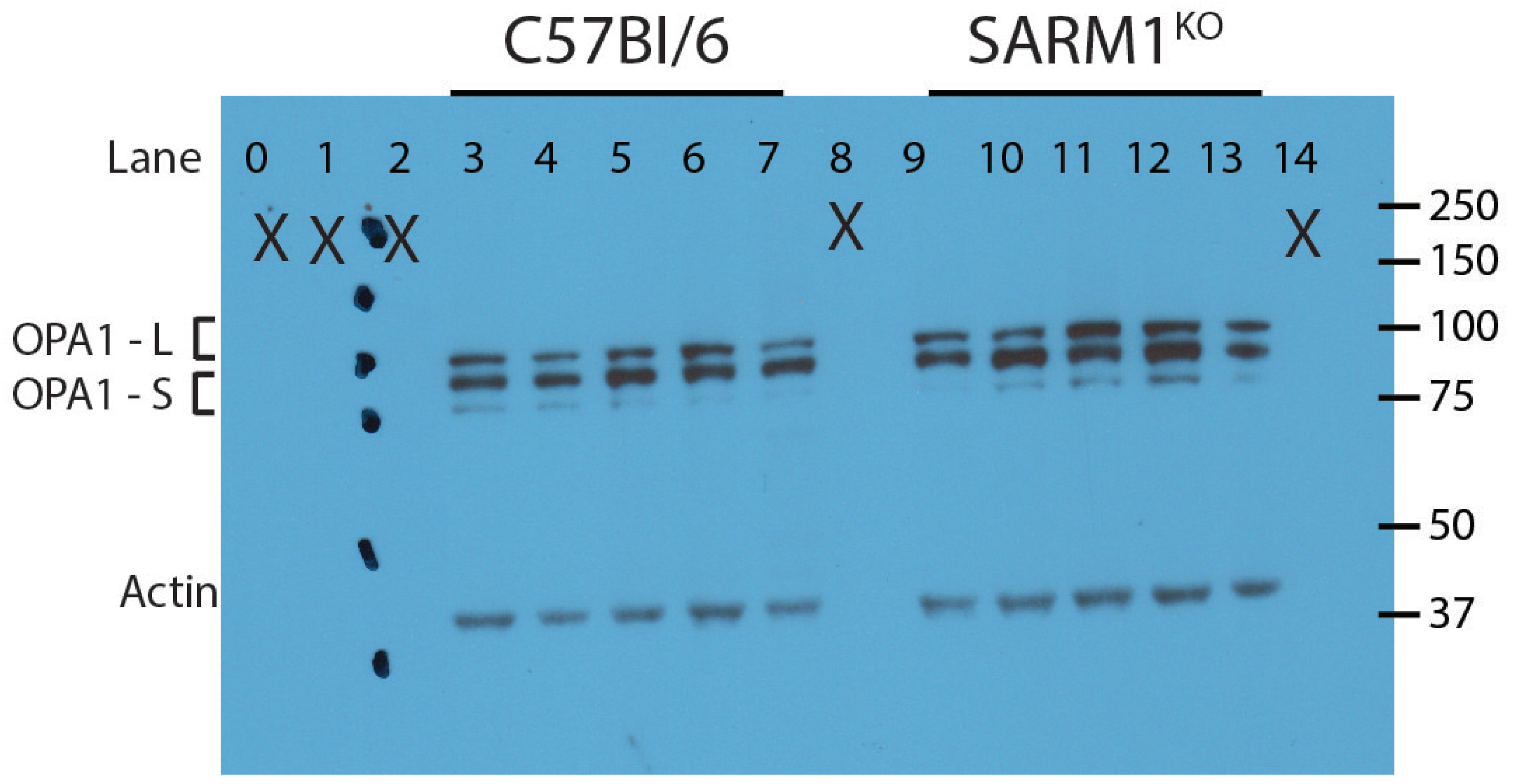

Panel A Far Right Inner Left

60 sec

NRF2 and Actin

C57Bl/6

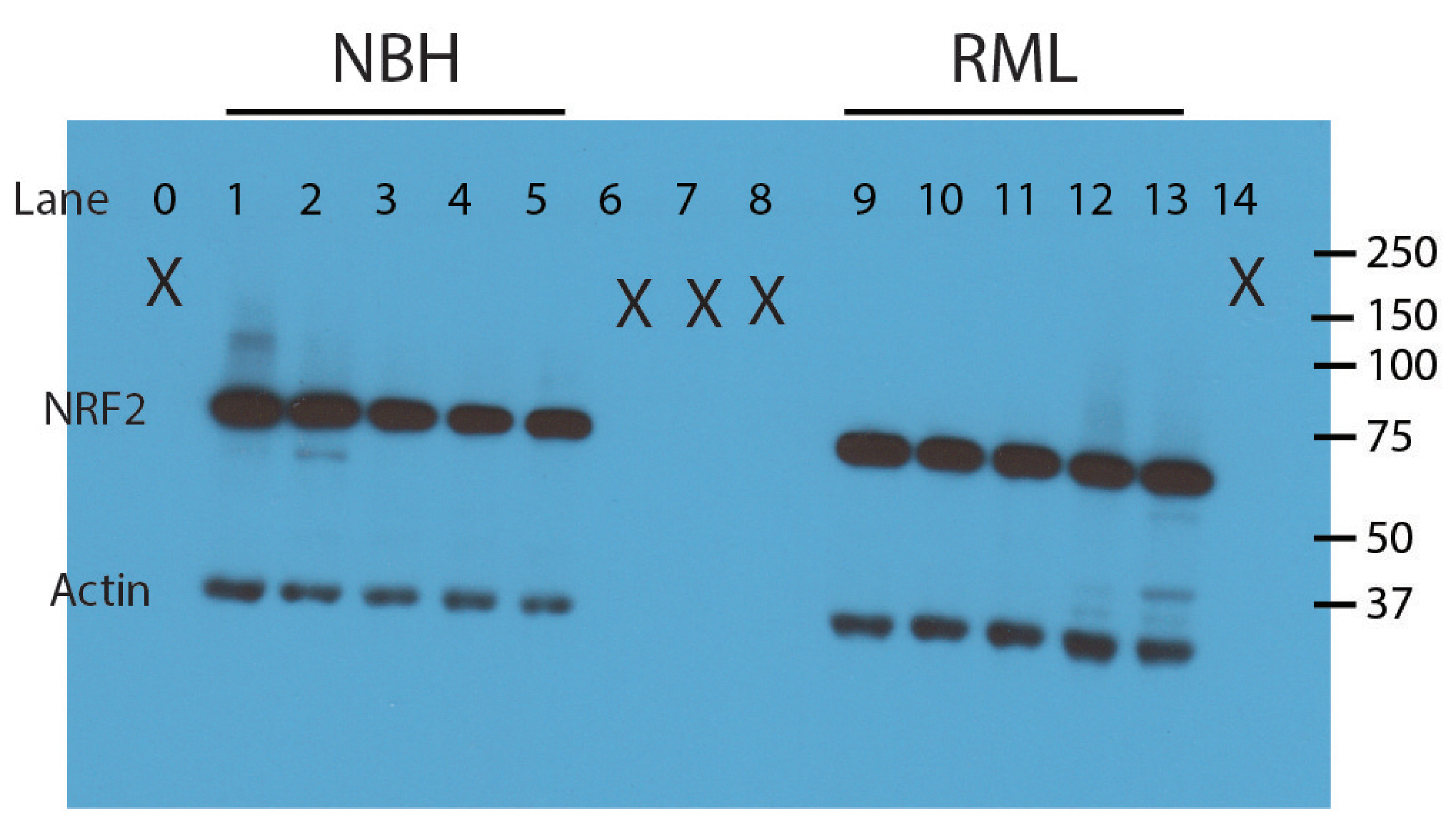

Panel A - Far / Inner Left 60 sec

SARM1KO

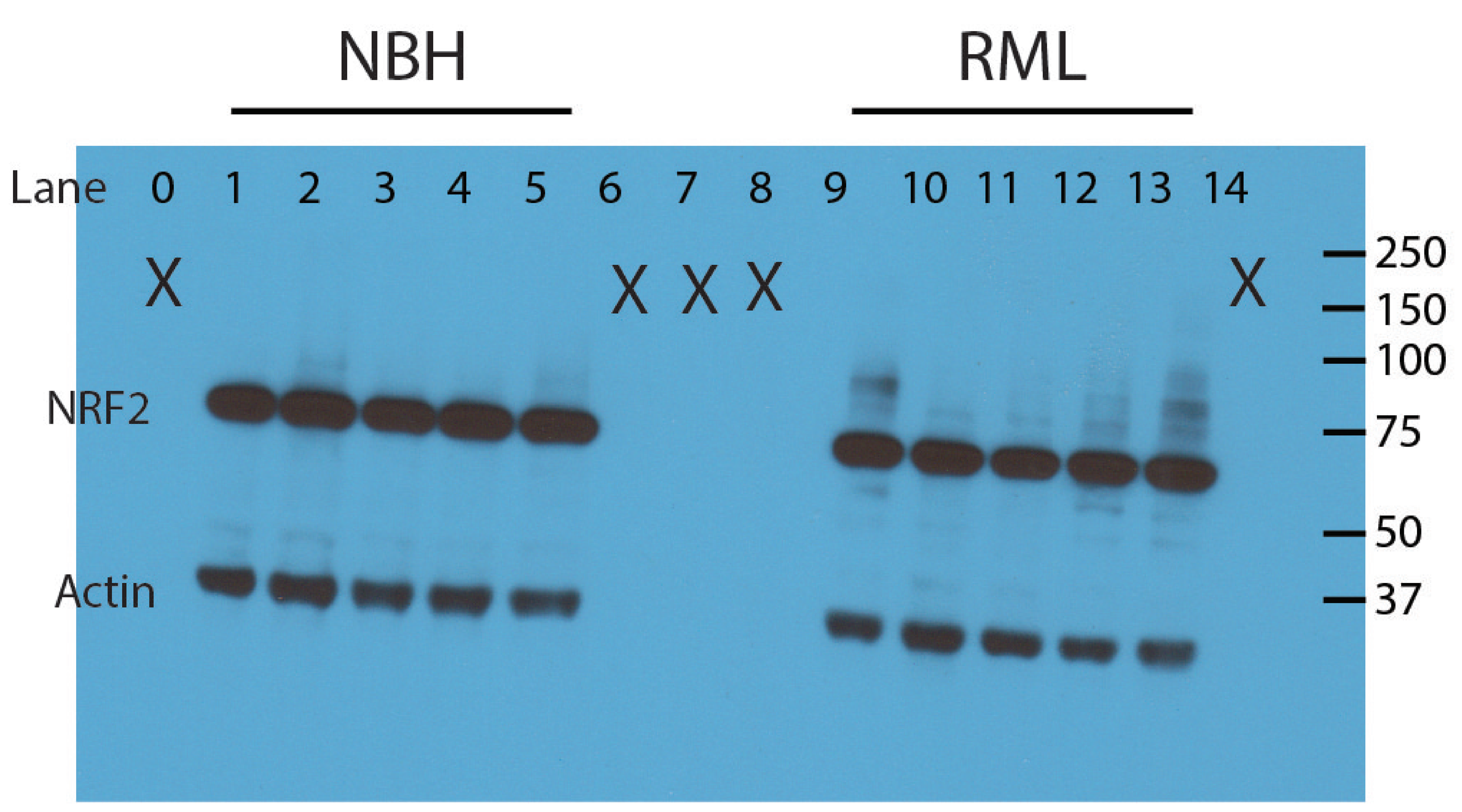

Panel A - Far / Inner Right 60 sec

Image captured using Epson Expression 10000 XL
Image converted to gray scale for final publication
